# Differences in neuroinvasion and protective innate immune pathways between encephalitic California Serogroup orthobunyaviruses

**DOI:** 10.1371/journal.ppat.1010384

**Published:** 2022-03-04

**Authors:** Alyssa B. Evans, Clayton W. Winkler, Karin E. Peterson

**Affiliations:** Laboratory of Persistent Viral Diseases, Rocky Mountain Laboratories, National Institute of Allergy and Infectious Diseases, National Institutes of Health, Hamilton, Montana, United States of America; University of Glasgow, UNITED KINGDOM

## Abstract

The California serogroup (CSG) of *Orthobunyaviruses* comprises several members capable of causing neuroinvasive disease in humans, including *La Crosse orthobunyavirus* (LACV), *Jamestown Canyon orthobunyavirus* (JCV), and *Inkoo orthobunyavirus* (INKV). Despite being genetically and serologically closely related, their disease incidences and pathogenesis in humans and mice differ. We have previously shown that following intraperitoneal inoculation of weanling mice, LACV was highly pathogenic while JCV and INKV were not. To determine why there were differences, we examined the ability of these viruses to invade the CNS and compared the host innate immune responses that regulated viral pathogenesis. We found that LACV was always neuroinvasive, which correlated with its high level of neuroinvasive disease. Interestingly, JCV was not neuroinvasive in any mice, while INKV was neuroinvasive in most mice. The type I interferon (IFN) response was critical for protecting mice from both JCV and INKV disease, although in the periphery JCV induced little IFN expression, while INKV induced high IFN expression. Despite their differing neuroinvasive abilities, JCV and INKV shared innate signaling components required for protection. The presence of either cytoplasmic Rig-I-Like Receptor signaling or endosomal Toll-Like Receptor signaling was sufficient to protect mice from JCV or INKV, however, inhibition of both pathways rendered mice highly susceptible to neurological disease. Comparison of IFN and IFN-stimulated gene (ISG) responses to INKV in the brains of resistant wild type (WT) mice and susceptible immune knockout mice showed similar IFN responses in the brain, but WT mice had higher ISG responses, suggesting induction of key ISGs in the brain is critical for protection of mice from INKV. Overall, these results show that the CSG viruses differ in neuroinvasiveness, which can be independent from their neuropathogenicity. The type I IFN response was crucial for protecting mice from CSG virus-induced neurological disease, however, the exact correlates of protection appear to vary between CSG viruses.

## Author summary

The California Serogroup (CSG) of Orthobunyaviruses contains several viruses that can cause encephalitis in humans, primarily in children, with different disease incidences. Mouse studies also showed differences between these viruses. *La Crosse orthobunyavirus* (LACV), the leading cause of pediatric arboviral encephalitis in the USA, is highly pathogenic in mice, while *Inkoo orthobunyavirus* (INKV) and *Jamestown Canyon orthobunyavirus* (JCV) caused only limited disease under certain conditions. In the current study, we analyzed the differences in neuroinvasion and host responses to these viruses. LACV entered the brain in all mice (highly neuroinvasive). INKV was able to invade the brain but was cleared and did not cause disease. JCV did not enter the CNS and was controlled in the periphery. Analysis of the early innate immune response showed that both INKV and JCV were controlled by type I interferon responses induced through either cytoplasmic or endosomal pattern recognition receptors. These studies show substantial differences between CSG viruses in their ability to invade the brain, cause neurological disease and the immune responses needed for the body to control these virus infections.

## Introduction

The California serogroup (CSG) of *Orthobunyaviruses* (family *Peribunyaviridae*) is a serologically and genetically related group of 18 known viruses [1]. All of the CSG viruses are mosquito-borne and some have been shown to cause neuroinvasive disease in humans, which primarly occurs in children [1–7]. Of the neuroinvasive CSG viruses, *La Crosse orthobunyavirus* (LACV) is found primarily in the USA and is responsible for the most neuroinvasive cases of CSG virsues annually [2,3]. *Snowshoe hare orthobunyavirus* (SSHV) causes a handful of neuroinvasive cases annually in the USA and Canada [4]. *Tahyna orthobunyavirus* (TAHV) is widely distributed throughout Europe, Africa and Asia and primarily causes febrile illness, but occasionally is neuroinvasive [1,5]. *Inkoo orthobunyavirus* (INKV) is mostly restricted to Scandinavia and has only been confirmed to cause a handful of neuroinvasive cases [6]. *Jamestown Canyon orthobunyavirus* (JCV) is responsible for an increasing number of neuroinvasive cases in the USA and Canada, and is the only CSG virus that primarily causes severe disease in adults [1,7].

We previously analyzed the pathogenesis of these five CSG viruses via intraperitoneal (IP) inoculation in C57BL/6 (B6) mice and found some similarities with the incidence and severity of disease in humans [8]. Consistent with previous studies, LACV was highly pathogenic in weanling mice [8–10], and SSHV and TAHV caused neuroinvasive disease in some or most weanling mice [8]. In contrast, JCV and INKV did not cause neuroinvasive disease in any weanling mice [8]. None of the viruses caused neuroinvasive disease in adult mice over the age of six weeks following IP inoculation [8]. However, when the peripheral immune system was bypassed and adult mice were inoculated intranasally (IN), LACV, SSHV, TAHV, and JCV all replicated extensively throughout the brain and caused neurological disease in nearly 100% of mice, indicating these CSG viruses are all highly neurovirulent [8]. The limiting factor for these viruses to cause neuroinvasive disease may therefore be the ability to gain access to the CNS. Interestingly, INKV only caused disease in ~25% of adult mice after IN inoculation and did not spread widely throughout the brains [8]. This indicates that INKV is only mildly neurovirulent and is likely controlled by immune responses within the brain, unlike the other CSG viruses. Determining the host immune responses involved in these differing CSG virus pathogenicities, in particular why JCV and INKV do not cause neuroinvasive disease in weanling mice, will further our understanding of CSG virus neuropathogenesis and the factors that mediate neurological disease.

One of the primary mechanisms that may limit virus spread within the periphery, to the CNS, and within the CNS is the type I interferon (IFN) system. Type I IFNs include several IFN classes, including multiple IFNα subtypes, as well as IFNβ, IFNε, and IFNκ [11]. The production of these IFNs is triggered by the recognition of viral pathogen associated molecular patterns (PAMPs) by either cytoplasmic RIG-I-like (RLR) receptors or the endosomal toll-like receptors (TLR) 3, 7, and 9 [11,12]. Virus activated RLRs signal through the mitochondrial antiviral-signaling (MAVS) protein, then the interferon regulatory response factors (IRF)3, IRF7, and IRF5 to activate *Ifn* mRNA transcription and production by a number of immune and non-immune cell types [13–15]. Similarly, the endosomal TLRs 3, 7, and 9 signal through the adaptor proteins MYD88 (TLR7 and 9) or TRIF (TLR3) and active IRF3, IRF7, and IRF5 to stimulate IFN production [15–18]. The multiple pathways of activation and multiple IRF transcription factors that are capable of inducing Type I IFNs leads to large amounts of redundancy in IFN production.

IFNs then bind to the IFN receptor (IFNAR) on cell surfaces to initiate a signaling cascade that results in the production of IFN stimulated genes (ISGs) with antiviral functions, including the IFN-induced proteins with tetratricopeptide repeats (IFITs) [11–14,16,19]. Previous studies have shown that the IFN response is important for protection during LACV infection and mediates LACV-induced age-dependent susceptibility to neuroinvasive disease in mice. Following IP inoculation, adult mice had a robust peripheral IFN response, primarily with *Ifna4* and *Ifn*β*1*, that was lacking in weanling mice, and this response mediated protection of adult mice from neuroinvasive disease [10,20]. Furthermore, it was determined that signaling through both cytosolic MAVS and endosomal TLR3/7/9 was required for protection of adult mice from neuroinvasive LACV disease, as adult mice deficient in either MAVS or TLR3/7/9 signaling were highly susceptible to neuroinvasive disease [10].

To directly investigate whether the differences in CSG virus pathogenesis were mediated by differences in neuroinvasive abilities and/or the IFN response, we inoculated weanling mice with 10^5^ PFU IP and conducted a time course to evaluate virus infection and IFN induction in peripheral tissues and the brain. We found that the highly pathogenic LACV invaded the brains of all mice, and the moderately pathogenic SSHV and TAHV invaded the brains of some, but not all mice. JCV did not enter the brains of any of the mice, suggesting control by peripheral immune responses. Despite not causing neuroinvasive disease, INKV gained access to the brains of most mice. Thus, INKV was neuroinvasive, but not neurovirulent, while JCV was not neuroinvasive. We further determined if there were IFN responses that correlated with neuroinvasion by examining IFN responses in the periphery to all viruses or neuropathogenesis by examining the IFN responses to INKV within the brains, as this is the only CSG virus controlled within the CNS. We also determined differences in the innate signaling pathways regulating CSG virus pathogenesis.

## Results

### The CSG viruses differ in neuroinvasion

**Fig 1:**
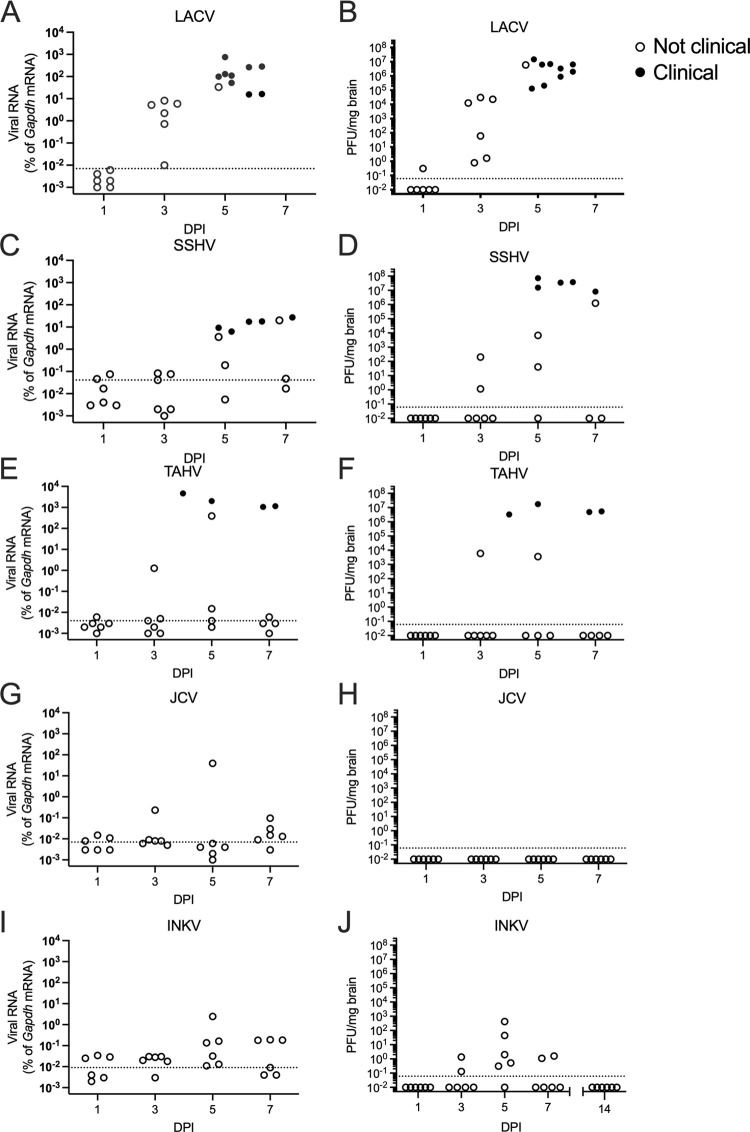
Neuroinvasion of the CSG viruses. A, C, E, G, I) Brain tissue was evaluated for viral RNA via RT-qPCR with virus-specific primers. Dotted lines indicate mock sample average from n = 4. B, D, F, H, J) Brain tissues were evaluated for infectious virus via plaque assays of brain homogenates in Vero cells. Dotted lines represent the limit of detection. No plaques were detected in any mock controls, n = 4. Individual dots represent individual mice. For all viruses and time points, n = 6, except for: LACV n = 4 for 6 dpi, no mice survived to 7 dpi; SSHV n = 5 for 5 dpi, two of the designated 7 dpi mice developed neurological disease at 6 dpi and had to be euthanized, so n = 2 for 6 dpi, n = 4 for 7 dpi; for TAHV, one mouse in the 5 dpi group developed neurological disease at 4 dpi and had to be euthanized, so n = 1 for 4 dpi and n = 5 for 5 dpi. (G) Dunnett’s multiple comparisons tests was done for JCV viral RNA compared to mock, using Log2(%gapdh) values. Nonclinical = mice with no signs of disease; Clinical = mice displaying neurological signs or other endpoint criteria as described in the methods.

To determine if the differences in pathogenesis of CSG viruses correlated with differences in the ability of the viruses to invade the CNS, we inoculated weanling mice intraperitoneally (IP) with 10^5^ PFU/mouse of the viruses and examined brain tissue at 1, 3, 5, and 7 dpi, or when a mouse showed neurological signs or other endpoint criteria. Viral RNA was evaluated via RT-qPCR with virus-specific primers, and infectious virus evaluated via plaque assay in Vero cells. LACV, the most neurovirulent of these viruses [8], had detectable viral RNA and infectious virus in the brains of all animals by 3 dpi which increased to higher levels at 5–6 dpi, the time points when mice developed clinical disease (Fig 1A and 1B). SSHV and TAHV, which were previously shown to cause neuroinvasive disease in some but not all mice at 10^5^ PFU/mouse [8], had a range of virus levels in the brains, with the highest levels observed in mice displaying neurological signs (Fig 1C and 1F). In the brains of some SSHV- and TAHV-inoculated mice that did not have neurological signs, viral RNA and PFUs were detectable at 3–7 dpi, although in others neither viral RNA or PFUs were detected (Fig 1C and 1F). The lack of virus in some mice at 5 and 7 dpi would suggest that one reason for the lower incidence of disease with SSHV and TAHV compared to LACV is a lack of virus entering the brain in some animals.

In previous studies, neither JCV nor INKV induced neurological disease following IP inoculation in weanling mice [8]. Surprisingly, these viruses showed divergent results for their abilities to enter the CNS. Although several JCV-inoculated mice had detectable viral RNA levels in the brain, none of the levels were significantly different from mock (Fig 1G). Additionally, no infectious virus was detected in any of the JCV-inoculated mouse brains (Fig 1H). The lack of virus in the brains suggests that JCV does not cause neurological disease following IP inoculation because the virus does not gain access to the CNS. In contrast, viral RNA and infectious virus were detected in brains for INKV-inoculated mice, with two of six mice at 3 dpi, five of six mice at 5 dpi, and two of six mice at 7 dpi having detectable PFUs in brain homogenates (Fig 1I and 1J). To determine if INKV was cleared from the brains after 7 dpi, an additional set of mice were inoculated with INKV and infectious virus analyzed at 14 dpi. No infectious virus was detected in any of these brains at 14 dpi (Fig 1J). Thus, INKV entered the brains of most weanling mice from the periphery, but was either unable to replicate sufficiently or was subsequently cleared by host immune responses in the brain prior to establishing a productive infection (Fig 1I and 1J).

### Virus in the periphery does not directly correlate with neuroinvasion

**Fig 2:**
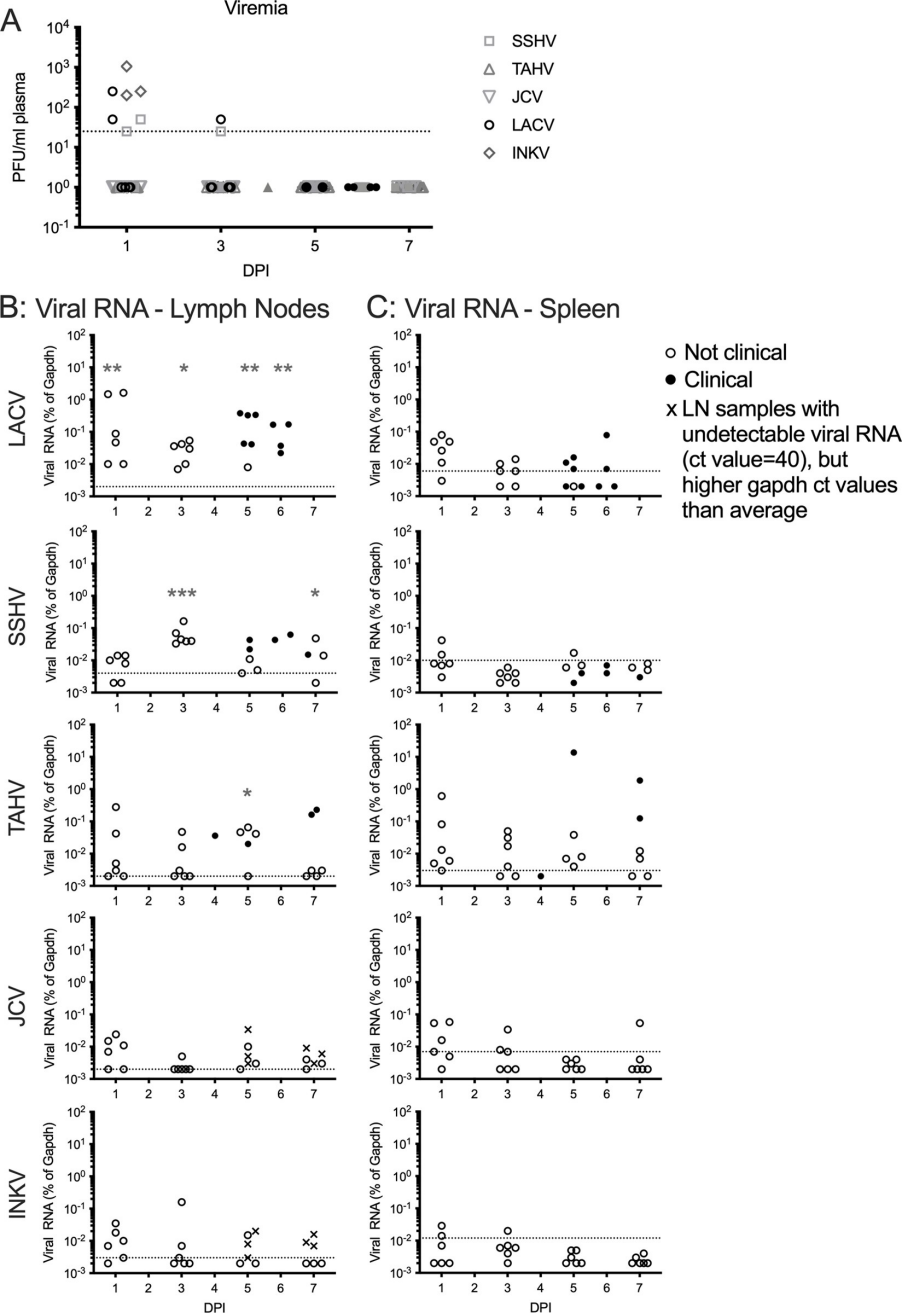
Virus in the periphery. A) Viremias were assessed via plaque assays of plasma samples in Vero cells. Dotted line represents the limit of detection. B-C) Viral RNA levels were analyzed via RT-qPCR with virus-specific primers in lymph nodes (B) and spleens (C). Dotted lines represent mock average from n≥3. Sample numbers are the same as described in Fig 1. Individual points represent individual mice. Dunnett’s multiple comparisons tests were done for each virus, tissue, and time point compared to mock, using Log2(%gapdh) values. Asterisks indicate sample days that had significantly higher viral RNA expression compared to mock. For mice that developed clinical signs prior to their designated time point, those values were grouped in with their designated time point (6dpi with 7dpi for SSHV, 4 dpi with 5 dpi for TAHV). Nonclinical = mice with no signs of disease; Clinical = mice displaying neurological signs or other endpoint criteria. *p = 0.05–0.01, **p = 0.009–0.001, ***p≤0.0009.

We next determined if the differences between CSG virus levels in the CNS correlated with differences in the amount of virus in the periphery by evaluating peripheral tissues over time from the same set of B6 mice used in the neuroinvasion studies. In plasma, infectious virus was not consistently detected for any of the CSG viruses (Fig 2A). Only 25% of LACV, SSHV, and INKV-inoculated mice had detectable viremias at 1 and/or 3 dpi, with no virus detected after 3 dpi (Fig 2A). JCV and TAHV-inoculated mice did not have detectable viremia in any plasma sample (Fig 2A). Viral RNA was observed in the inguinal lymph nodes (LN) of most LACV-, SSHV-, and TAHV-inoculated mice, but in only a few JCV- and INKV-inoculated mice (Fig 2B). However, only LACV-inoculated mice had viral RNA expression significantly higher than mock on all dpi, while viral RNA was only significantly different from mock for SSHV at 3 and 6/7 dpi and TAHV at 5 dpi (Fig 2B). No significant viral RNA was detected in the LN for JCV- or INKV-inoculated mice at any dpi (Fig 2B).

Viral RNA was low to undetectable in the spleens of all mice inoculated with LACV, SSHV, JCV, or INKV (Fig 2C). TAHV-inoculated mice had detectable viral RNA over the time course, however this expression was not significantly different from mock (Fig 2C). Overall, these results suggest that none of the CSG viruses replicated extensively in the periphery. Furthermore, there was not a strong correlation between virus infection in the periphery and virus infection in the CNS. The neuroinvasive viruses (LACV, SSHV, TAHV, and INKV) were not remarkably higher in the plasma or spleens compared to the non-neuroinvasive JCV. However, the neuropathogenic viruses (LACV, SSHV, and TAHV) did appear to have higher viral RNA levels in the LNs than either of the non-neuropathogenic viruses (JCV and INKV; Fig 2B).

### CSG viruses differ in peripheral interferon response

From previous LACV studies we know that age-dependent resistance to neuroinvasive disease is due, in part, to a strong type I IFN response in adult mice that is lacking in weanling mice [10]. Therefore, we evaluated if there were differences in IFN responses between the CSG viruses that would help explain differences in their neuroinvasiveness and neuropathogenicity in weanling mice. To do this, we evaluated the IFN response in the periphery by performing RT-qPCR on RNA isolated from the inguinal LNs and spleens from the same time course of B6 weanling mice described above. From our analysis of viral RNA in the periphery, we determined that overall the LNs had more detectable viral RNA than spleens for the CSG viruses (Fig 2B and 2C). Most of the CSG viruses had detectable to high levels of virus in the LN at 1 dpi, therefore we analyzed mRNA expression of 10 type 1 IFNs in the 1 dpi LNs for all viruses to identify the IFN transcripts induced in response to the CSG viruses.

**Fig 3:**
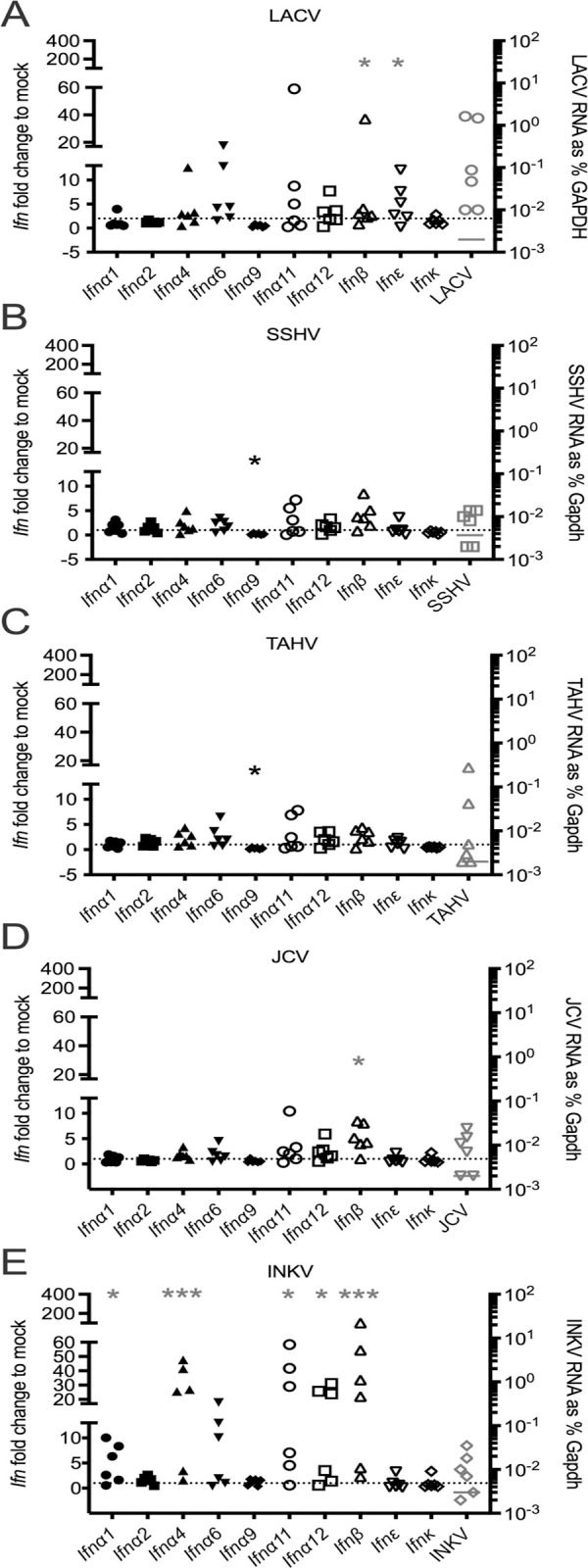
IFN response to CSG viruses in 1 dpi lymph nodes. IFN mRNA expression was evaluated via RT-qPCR for a panel of 10 Type I IFNs in lymph nodes taken at 1 dpi from mice inoculated with A) LACV; B) SSHV; C) TAHV; D) JCV; E) INKV. Expression is plotted for each samples as the fold change in %gapdh from the mock average. N = 6 for all viruses, n = 7 for mock. Each individual point represents an individual mouse. Dotted lines indicate fold change = 1. Fold change to mock is plotted on the left axis, viral RNA plotted on the right axis. One-way ANOVA analyses were performed on Log2(%gapdh) with Dunnett’s multiple comparison test performed between mock and each virus. Asterisks denote *Ifn* expression that was significantly different from mock for that IFN and virus (gray = significantly higher, black = significantly lower than mock): *p = 0.05–0.01, **p = 0.009–0.001, ***p≤0.0009.

Several IFN mRNAs were significantly increased in the LN following virus infection, although the IFN type and expression level varied between viruses. *Ifn*β*1* was significantly upregulated by LACV, JCV, and INKV infection and *Ifnε* by LACV infection (Fig 3A, 3D and 3E). Of the IFN alpha subtypes, *Ifna1*, *Ifna4*, *Ifna11*, and *Ifa12* mRNA expression was significantly increased during INKV infection (Fig 3E), but not with the other viruses. Indeed, the only IFN mRNA with significantly different expression from mock in 1 dpi LNs from SSHV and TAHV-infected mice was *Ifna9*, which was lower than mock levels (Fig 3B and 3C).

Expression of *Ifna4*, *Ifna11*, *Ifna12*, *Ifn*β*1*, and *Ifnε* mRNAs was further analyzed in the LNs at 3 dpi and spleens at 1 dpi for all viruses. The full panel of 10 IFN mRNAs was also analyzed for SSHV at 3 dpi and TAHV at 4/5 dpi, because these were the days when these viruses had the highest level of viral RNA in the LNs. By 3 dpi, the IFN mRNA response in the LNs had almost entirely resolved for most mice infected with LACV, JCV, and TAHV, and the low levels were maintained for TAHV at 4/5 dpi (S1 Fig). INKV-infected mice still had increased *Ifna4* and *Ifn*β*1* mRNA in the LN at 3 dpi, but lower than levels at 1 dpi (Figs S1 and 3E). SSHV-infected mice had increased *Ifn*β*1* mRNA in the LNs at 3 dpi (S1A Fig), which corresponded with the increase in viral RNA observed in the LN at 3 dpi (Fig 2). No significant upregulation in IFN mRNA expression was observed in the spleen at 1 dpi for any virus (S1C Fig). Overall, these results show that INKV had the highest and most robust IFN mRNA response in the periphery of any of the CSG viruses, with five IFN mRNAs significantly upregulated in the LNs at 1 dpi and two at 3 dpi (Figs 3E and S1A). LACV had an intermediate peripheral IFN mRNA response with two IFN mRNAs significantly upregulated in the LNs at 1 dpi, but three additional IFN mRNAs trending higher (Fig 3A). SSHV, TAHV and JCV had a low IFN mRNA response, with only one IFN mRNA significantly upregulated for SSHV (in the LN at 3 dpi) and JCV (in the LN at 1 dpi), and none significantly upregulated for TAHV (Figs 3 and S1). However, it is unclear if the increased IFN mRNA response observed in INKV was protective, as the virus appeared to be able to evade this response and gain access to the CNS.

### The IFN and ISG responses to INKV and LACV are similar in the brain

**Fig 4:**
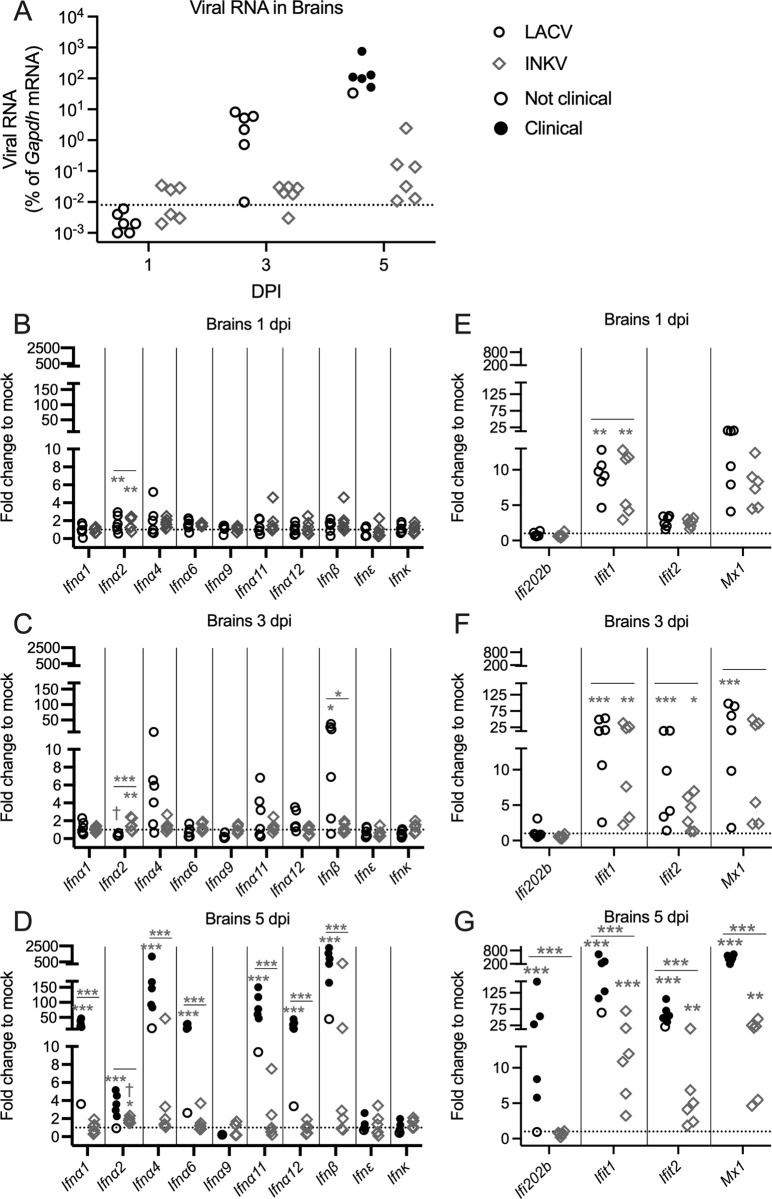
IFN response to LACV and INKV in the brains. A) Viral RNA values are the same as Fig 1A and 1I, but are represented here for a direct comparison of virus in LACV-infected and INKV-infected mice at 1, 3, and 5 dpi. IFN mRNA expression of 10 IFNs in the brains of LACV- and INKV-infected mice at (B) 1 dpi, (C) 3 dpi, and (D) 5 dpi. ISG mRNA expression in the brain was compared between LACV- and INKV-infected mice at (E) 1 dpi, (F) 3 dpi, and (G) 5 dpi. B-G) Dotted lines indicate fold change = 1. One-way ANOVA analyses were performed on Log2(%gapdh) values comparing the expression of each individual IFN and ISGs for mock (n≥4), LACV (n = 6), and INKV (n = 6), with Dunnett’s multiple comparison test performed between mock and each virus, and follow-up analyses with Sidak’s multiple comparsions test between LACV and INKV at any day with any IFN or ISG mRNA expression levels significantly higher than mock. Asterisks below the line represent expression significantly different from mock for that virus and IFN or ISG, and asterisks above the line represent a significant difference between LACV and INKV for that IFN or ISG mRNA at that dpi. If no asterisks are present above the line, this indicates there was no significant difference. The † represents significantly higher expression of *Ifna2* between INKV at 5 dpi and LACV at 3 dpi (p<0.0001). *p = 0.05–0.01, **p = 0.009–0.001, ***p≤0.0009. Individual points represent individual mice. Nonclinical = mice with no signs of disease; Clinical = mice displaying neurological signs or other endpoint criteria.

A strong IFN response to INKV infection in the CNS could be a mechanism by which INKV is controlled within the brain. Therefore, we compared type I IFN mRNA expression in brain tissue from the INKV and LACV-inoculated mice at 1, 3, and 5 dpi. These were the same mice as described above. We did not include 6 dpi for LACV because all of these mice had indistinguishable virus levels from the 5 dpi mice (Fig 1A and 1B). As IFN expression can be a direct response to the level of virus, we also compared IFN responses between brains from nonclinical INKV-infected mice at 5 dpi and pre-clinical LACV-infected mice at 3 dpi because these time points had the most similar viral levels (Figs 1A, 1B, 1I, 1J and 4A). SSHV and TAHV were not included in the analysis because the wide variation in virus loads at different time points would make it difficult to interpret findings (Fig 1C–1F). JCV was not included because no infectious virus was detectable in the brains (Fig 1H).

Overall, there was lower expression of IFN mRNA in response to INKV infection in the brain than what was observed in the periphery (Figs 4B–4D and 3E). In INKV-inoculated mice, the only IFN mRNA that was statistically increased from mock levels was *Ifna2*, which was elevated at 1–5 dpi (Fig 4B–4D), but went back basal levels by 7 dpi (S2A Fig). Interestingly, *Ifna2* mRNA in brains of INKV-infected mice at 3 dpi and 5 dpi was significantly higher than in LACV-infected mice at 3 dpi (Fig 4C and 4D), despite INKV-infected mice having similar (5 dpi) or lower (3 dpi) levels of virus in the brains. Several IFN mRNAs, particularly *Ifna2*, *Ifna4*, *Ifna11*, and *Ifn*β*1*, appeared to be elevated in brains of LACV-infected mice at 1–3 dpi (Fig 4). However, statistical analysis showed that only *Ifna2* mRNA at 1 dpi and *Ifn*β*1* mRNA at 3 dpi were significantly different from mock-inoculated mice (Fig 4B and 4C). At 3 dpi, only *Ifn*β*1* mRNA was significantly higher in brains of LACV-inoculated mice compared to INKV-infected mice (Fig 4C). At 5 dpi, *Ifna1*, *Ifna2*, *Ifna4*, *Ifn6*, *Ifna11*, *Ifna12*, and *Ifn*β*1* mRNA levels were significantly increased in brains of LACV-infected mice compared to mock-inoculated controls (Fig 4D) and expression of all but *Ifna2* were significantly increased compared to INKV-inoculated mice (Fig 4D). This robust IFN response at 5 dpi in LACV reflected the high level of virus in the brains and was clearly not protective, as five of the six LACV-inoculated mice had neurological disease (Figs 1B and 4A). Overall, LACV and INKV had similar levels of IFN expression in the brains up until 5 dpi, when most LACV-inoculated mice developed neurological disease.

We next evaluated whether ISG responses to LACV and INKV differed in the brains of these same weanling mice. We examined *Ifit1* and *Ifit2*, which have been shown to respond to multiple viral infections in the brain [19,21]; *Ifi202b*, which has previously been shown to be upregulated by microglia following LACV and other viral infections in the brain [22]; and *Mx1*, which has been shown to restrict LACV growth in vitro and in transgenic mice, although the protein is non-functional in WT B6 mice [23,24]. mRNA expression of these four ISGs was analyzed over the time course via RT-qPCR. Surprisingly, at 1 dpi *Ifit1* and *Mx1* mRNA expression was elevated from mock in brains of both LACV and INKV-inoculated mice, with a significant increase in *Ifit1* mRNA compared to mock (Fig 4E). At 3 dpi, *Ifit1*, *Ifit2*, and *Mx1* mRNA were elevated in brains of LACV and INKV-inoculated mice, and all were significantly different from mock except *Mx1* mRNA in INKV-inoculated mice (Fig 4F). All four ISG mRNAs were highly expressed in brains of LACV-inoculated mice at 5 dpi (Fig 4G). *Ifit1*, *Ifit2*, and *Mx1* mRNA expression remained high in brains of INKV-inoculated mice at 5 dpi as well, with all three mRNAs significantly increased compared to mock controls, but also significanly lower than in LACV-inoculated mice (Fig 4G). None of the ISG mRNA expression levels were significantly different between brains of LACV-inoculated mice at 3 dpi and INKV-inoculated mice at 5 dpi, where viruses levels were the most similar (Fig 4A). These results suggest that CSG viruses, particularly INKV, can induce a robust ISG response from a small or moderate type I IFN response. However, at least in the case of LACV, where all mice develop neurological disease by 6 dpi, this IFN response in the CNS was not sufficient for protection.

### Immune cell reactivity in LACV- and INKV-infected brains corresponds with neuroinvasion

**Fig 5:**
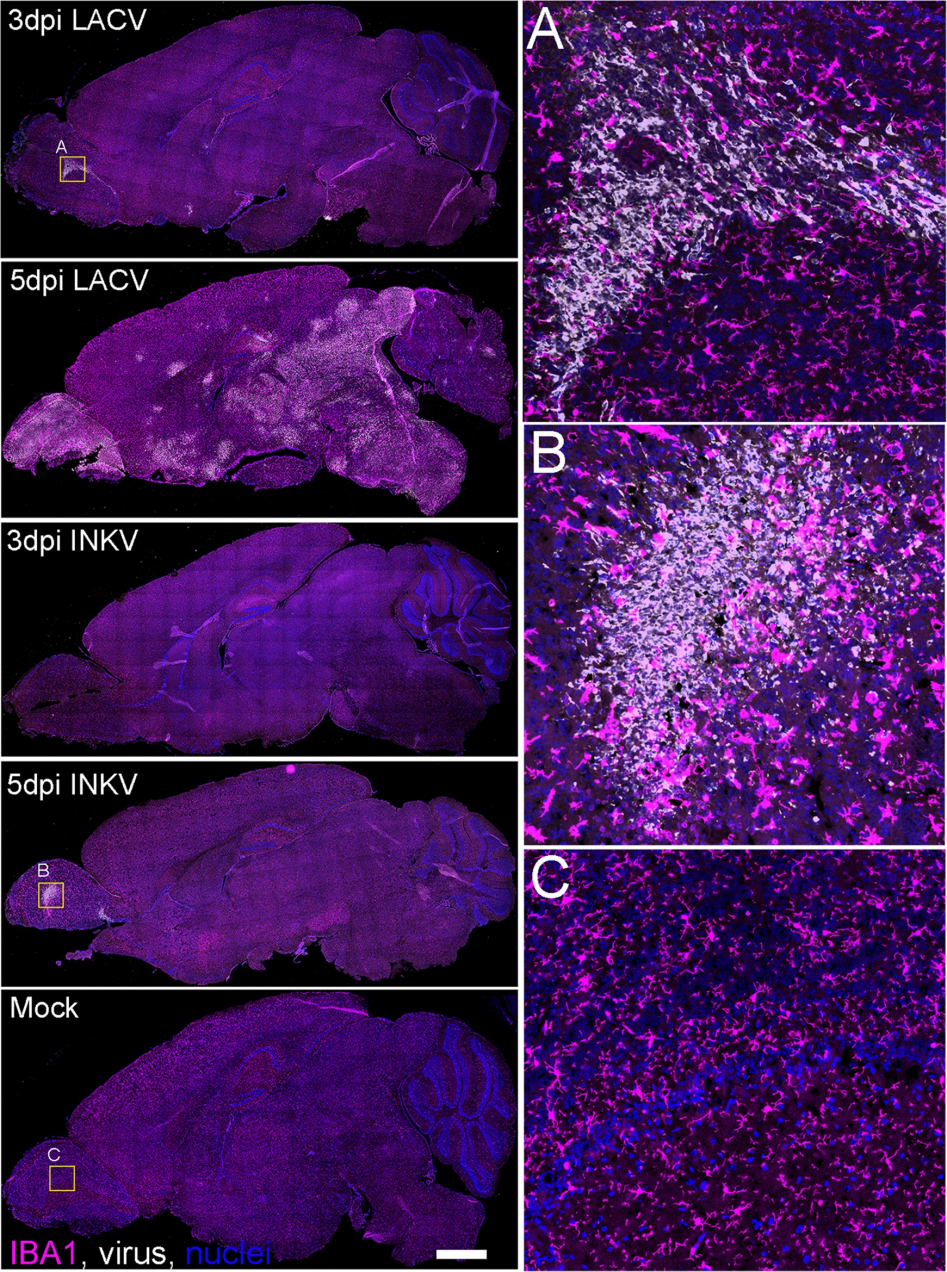
Immune responses to LACV and INKV infection in the brain. Representative whole-brain sagittal sections from (left column, top to bottom), 3 dpi LACV, 5 dpi LACV, 3 dpi INKV, 5 dpi INKV and mock-inoculated mice were immunohistochemically labeled for the myeloid/microglia-specific marker ionized calcium binding adaptor molecule 1 (IBA1, magenta), virus (white) and cell nuclei (blue). The white scale bar in the mock-inoculated panel represents 1mm and corresponds to all other images. The yellow boxes in the 3 dpi LACV (A), 5 dpi INKV (B), and mock-inoculated (C) sections correspond to high magnification insets shown in the right column highlighting the myeloid cellular morphology in the olfactory bulb of each respective animal.

We next examined if there were differences in microglial activation or leukocyte recruitment within the brains of LACV- and INKV-inoculated mice. Weanling B6 mice were inoculated IP, brains were removed at 3 and 5 dpi, and half used for fluorescent immunohistochemistry (IHC) and the other half used for plaque assays. For IHC, frozen sections were stained for virus antigen, the myeloid/microglia-specific marker ionized calcium binding adaptor molecule 1 (IBA1), and the T cell-specific marker CD3. Full brain sections were scanned as described in the methods and the resulting images were blindly scored for virus and microglial activation as determined by IBA1+ labeling and cell morphology (Table 1 and Fig 5). These data were correlated with plaque assay data obtained from the contralateral hemisphere (Table 1).

**Table 1:** Scoring of Virus, Microglial Activation, and Perivascular Cuffing from Immunohistochemistry.

			Virus			IBA1+ Microglial Activation		Perivascular Cuffing	
Virus	DPI	Mouse ID	Titer PFU/ml	IHC Score	Location	Activation Score	Location	Present?	Location
LACV	3	**297–1** *	**1.14E+03**	**2**	**OB, ON, HT, T, M**	**3**	**OB, ON, HT, T, M, C**	**Yes**	**T**
		297–2	0	0		2	OB, S, C	No	
		296–1	1.97E+03	2	P	3	P, HC	Yes	P
		Average:	1.04E+03	1.3		2.7			
	5	**296–2** *	**4.23E+07**	**5**	**OB, ON, S, T, C, MB, CB, P, M, HC, CP, HT**	**5**	**OB, ON, S, T, C, MB, CB, P, M, HC, CP, HT**	**Yes**	**C, CP, T, P, HT, OB**
		296–3	2.53E+07	5	OB, ON, S, T, C, MB, CB, P, M, HC, CP, HT	5	OB, ON, S, T, C, MB, CB, P, M, HC, CP, HT	Yes	ON, C, CP, T, S, HT, P, MB
		296–4	1.05E+07	5	OB, ON, S, T, C, MB, CB, P, M, HC, CP, HT	5	OB, ON, S, T, C, MB, CB, P, M, HC, CP, HT	Yes	ON, C, CP, T, S, HT, P
		Average:	2.60E+07	5		5			
INKV	3	298–1	0	0		0		No	
		**298–2** *	**0**	**0**		**0**		**No**	
		298–3	0	0		0		No	
		Average:	0	0		0			
	5	301–2	4.87E-01	0		2	S, T AON, C	Yes	AON, S
		302–3	4.01E+02	0		2	HT, HC, MB, P	Yes	HT, S
		302–2	1.36E+00	1	OB, MB	2	HT, T, S, MB	Yes	S, CP
		**301–4** *	**3.38E-01**	**2**	**OB, AON, CB**	**3**	**OB, AON, S, HT, MB, CB**	**Yes**	**AON, MB, S**
		302–1	0	0		2	OB, S, P, M	Yes	S, MB
		301–3	0	0		0		No	
		Average:	7.E+01	0.5		1.8			
Mock	3/5	**293–1** *	**0**	**0**		**1**	**T**	**No**	
		295–1	0	0		0		No	
		300–1	0	0		0		No	
		Average:	0	0		0.3			
	Virus Scoring			IBA1+ Microglial Activation Scoring			Abbreviations		
Scoring Criteria	0-no obvious virus labeling 1-patches of <10 cells, <1% brain area 2-patches of ≥10 cells, 1–5% brain area 3-large patches of cells, <20% brain area 4-merged patches of cells, <50% brain area 5-extensive virus labeling, ≥50% brain area			0-no obvious IBA1+ cell activation 1-patches of <10 IBA1+ activated cells 2-patches of ≥10 IBA1+ activated cells 3-large patches of cells, <20% brain area 4-merged patches of cells, <50% brain area 5-extensive IBA1+ labeling, ≥50% brain area			P = pons MB = midbrain S = striatum HT = hypothalamus CB = cerebellum OB = olfactory bulb C = cortex ON = olfactory nucleus T = thalamus CP = caudate putamen M = medulla AON = ant. olfactory nucleus HC = hippocampus		

*Bold samples shown in Fig 5

For LACV-inoculated mice, two out of three brains at 3 dpi and three out of three brains at 5 dpi had detectable virus plaques, with titers corresponding to the IHC viral antigen score (Table 1). For the two virus-positive 3 dpi LACV brains, viral antigen staining was in small, discrete patches in the pons, olfactory bulb (Fig 5A), olfactory nucleus, hypothalamus, thalamus, and/or the medulla (Table 1). The 5 dpi LACV brains from mice with neurological signs had widespread virus throughout most of the brain (Table 1 and Fig 5). For INKV, none of the three brains at 3 dpi had detectable plaques or viral antigen (Table 1 and Fig 5). At 5 dpi, plaque assay titers did not directly correspond with virus antigen staining for INKV brains. Four out of the six mice had detectable plaques in the brains, but only two of these had detectable viral antigen staining (Table 1). In the two brains with detectable INKV antigen, the virus was only found in small patches in the olfactory bulb (Fig 5C), midbrain, anterior olfactory nucleus, or the cerebellum (Table 1). The discrete virus antigen foci observed in these sections correlated with the lack of viral antigen staining in some tissue sections from mice with detectable infectious INKV in the brains. The foci were scattered within the brain of INKV-inoculated mice, and therefore were likely missed in some mice. It is of interest that in the two INKV brains with detectable viral antigen, INKV was identified in the olfactory bulb of both animals. Previous studies of LACV have shown that it enters the brain via capillary breakdown specifically in the olfactory bulb [25], and these data suggest INKV may enter the brain in a similar fashion. No viral antigen was detected in any mock-inoculated mouse brain (Fig 5C).

The amount of microglial activation and the presence of perivascular cuffing generally correlated with the amount of virus present in the brain tissue. Brains with higher virus titers by plaque assay tended to have higher scores for microglial activation (Table 1). For example, the 3 dpi brains from INKV-inoculated mice had no microglial activation, and the 5 dpi brains from LACV-inoculated mice had high amounts of microglial activation that occurred in the same brain regions as the virus (Table 1 and Fig 5). Brains from LACV-inoculated mice at 3 dpi and INKV-inoculated mice at 5 dpi had more variable microglial activation, but mostly corresponded to virus staining. Indeed, microglial activation was detected in all of the brain regions where virus antigen was present, except the olfactory bulb for INKV mouse 302–2 at 5 dpi, but was also detected in at least one additional brain region without detectable viral antigen (Table 1). Additionally, all of the plaque-positive INKV brains at 5 dpi, regardless of virus antigen staining, had detectable microglial activation. The mock-inoculated brains had IBA1 staining throughout the brains that looked morphologically unreactive, except for two cells from one section (Table 1 and Fig 5).

Analysis of CD3 showed few, if any, CD3-positive cells in any brains from mock-inoculated controls, and only slightly more CD3-positive cells in brains from INKV- or LACV-inoculated mice at any time point (S3 Fig). This was not surprising as we have previously shown T-cell recruitment to the brain is limited in clinical LACV disease and does not play a role in pathogenesis [26,27]. Overall, there was no striking difference in virus distribution or immune cell reactivity during early stages of brain infection for either LACV-inoculated (3 dpi) or INKV-inoculated (5 dpi) mice.

### The interferon response protects mice from JCV and INKV-induced neuroinvasive disease

**Fig 6:**
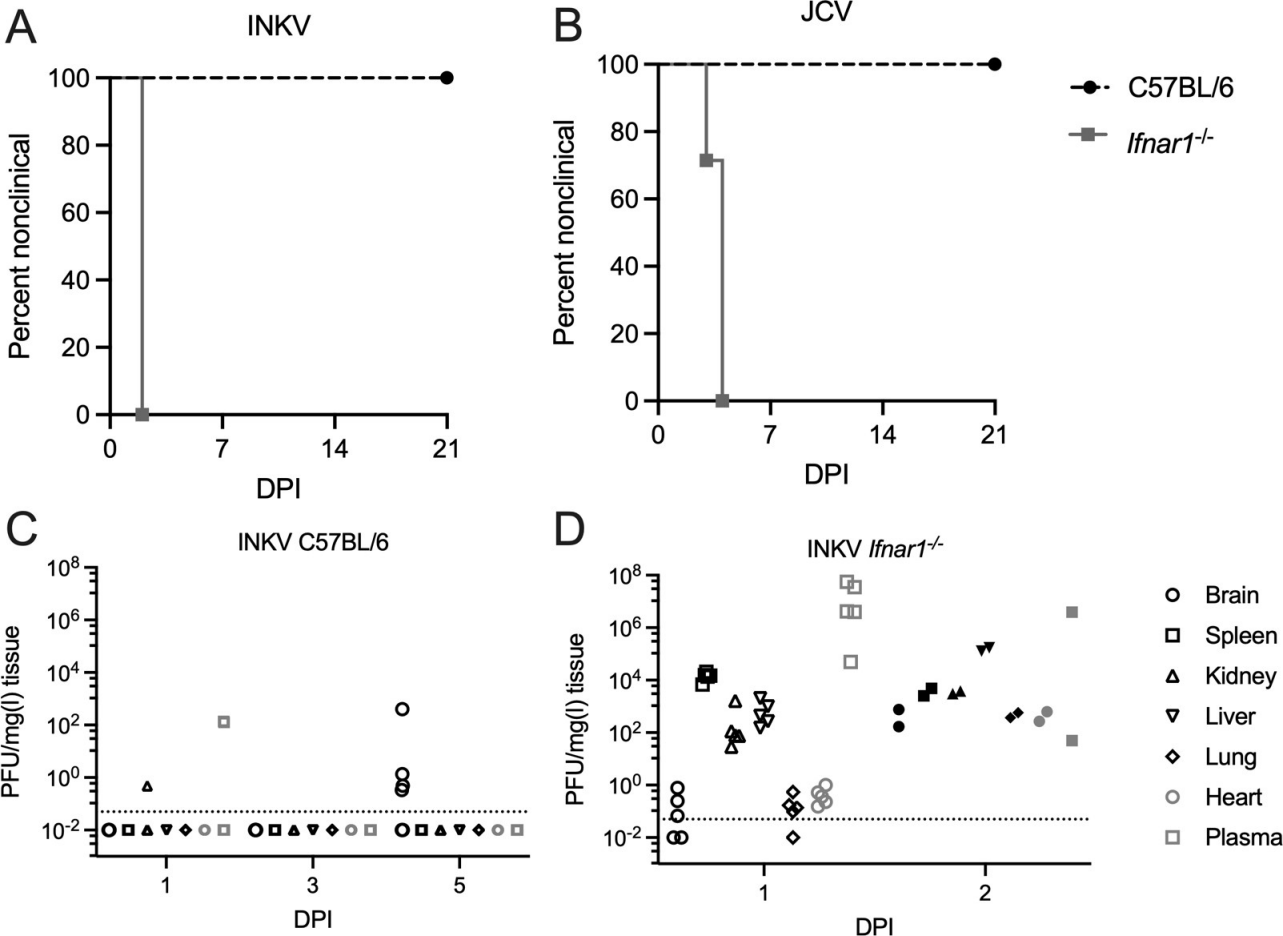
Disease curves and virus loads for C57BL/6 mice and *Ifnar1*^-/-^ mice. Mice were inoculated IP with 10^5^ PFU of A) INKV, n = 6 for C57BL/6, n = 9 for *Ifnar1*^-/-^ or B) JCV, n = 5 for C57BL/6, n = 7 for *Ifnar1*^-/-^, and followed for neurological signs, other endpoint criteria, or death. Timepoint analysis of infectious virus via plaque assay of tissue homogenates in Vero cells of tissues from INKV-inoculated C) WT B6 mice and D) *Ifnar1*^-/-^ mice.

The results from IFN mRNA expression analysis did not yield obvious results as to which IFN may mediate protection, or if the IFN response is involved in protecting mice from JCV- and INKV-induced neuroinvasive disease at all. Therefore, to determine if the IFN response had a direct role in protecting weanling mice from JCV- and INKV-induced neuroinvasive disease, we inoculated *Ifnar1*^*-/-*^ knockout mice, which lack a functional type I IFN receptor, with either JCV or INKV and evaluated the mice for clinical signs. INKV-inoculated *Ifnar1*^*-/-*^ mice died at 2 dpi, while JCV-inoculated *Ifnar1*^*-/-*^ mice all developed neurological disease or died at 3–4 dpi (Fig 6A and 6B).

While the mice inoculated with JCV showed clear neurological signs of ataxia and hind limb weakness, the INKV-inoculated mice died suddenly at 2 dpi, making it impossible to determine if they had neurological signs. We therefore inoculated additional *Ifnar1*^*-/-*^ and B6 mice with INKV and analyzed infectious virus levels in tissues from five mice each at 1 dpi for both strains, two moribund mice at 2 dpi for *Ifnar1*^*-/-*^, and six nonclinical mice at 3 and 5 dpi for B6. Infectious virus was measured in brains, spleens, kidneys, livers, lungs, hearts, and plasma via plaque assay in Vero cells. From all of the INKV-inoculated B6 mice, one kidney and two plasma samples at 1 dpi as well as four brains at 5 dpi had detectable plaques (Fig 6C). In contrast, at 1 dpi *Ifnar1*^*-/-*^ mice all had high levels of virus in multiple tissues including spleen, kidney, liver, lungs, brains, and plasma (Fig 6D). The two moribund INKV-inoculated *Ifnar1*^*-/-*^ mice at 2 dpi that tissues could be taken from both had high levels of virus in all tissues (Fig 6D). These results demonstrate that a functional type I IFN response has a crucial role in protecting mice from widespread viral dissemination and death following infection with INKV, and the development of neurological disease with JCV.

### CSG viruses differ in the innate immune pathway components involved in protection of mice from neuroinvasive disease

We next examined the pathways and components of the upstream innate immune system involved in generating the IFN response. Previous studies of LACV infection showed that both endosomal toll-like receptors (TLRs) and cytoplasmic Rig-I like receptors (RLRs) signaling pathways were necessary for protecting adult mice from neuroinvasive disease. Therefore, we inoculated JCV and INKV IP into mice deficient in key components of these pathways including *Irf3*^*-/-*^*xIrf7*^*-/-*^ double knockout (DKO) mice, *Myd88*^-/-^ mice which lack the adaptor protein for most TLRs other than TLR3, *Unc93b1*.3D mice that lack functional endosomal TLRs, and *Mavs*^-/-^ mice that lack functional RLR signaling. Mice were followed for the development of neurological disease, which primarily consisted of ataxia and hind limb weakness/paralysis, and occasionally tremors, circling, and/or seizures.

**Fig 7:**
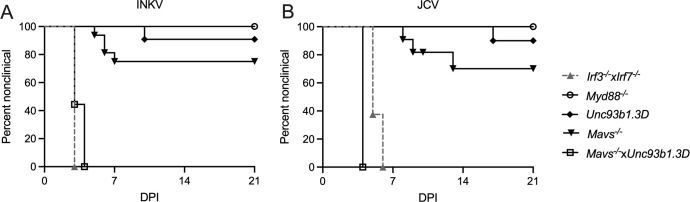
Disease curves in immune deficient mouse strains. Mice were inoculated IP with 10^5^ PFU of A) INKV or B) JCV, and followed for neurological signs, other endpoint criteria, or death. For *Irf3*^*-/-*^*xIrf7*^*-/-*^ n = 8 INKV and JCV; for *Myd88*^*-/-*^ n = 5 INKV, n = 8 JCV; for *Unc93b1*.3D n = 11 INKV, n = 10 JCV; for *Mavs*^*-/-*^ n = 14 INKV, n = 10 JCV; for *Mavs*^*-/-*^*xUnc93b1*.3D n = 9 INKV, n = 10 JCV.

All weanling *Irf3*^-/-^x*Irf7*^-/-^ DKO mice either died or developed neurological disease at 3 dpi for INKV, and at 5–6 dpi for JCV, indicating that signaling through IRF3 and/or IRF7 was critical for the protection of mice from neuroinvasive disease from JCV and INKV (Fig 7A and 7B). However, knockouts in individual pathway components resulted in surprising results in infected mice. Only 25% of *Mavs*^-/-^ mice and less than 10% of *Unc93b1*.3D mice developed clinical signs following JCV or INKV inoculation, while none of the *Myd88*^-/-^ mice showed signs of disease (Fig 7A and 7B). To determine if mice would be susceptible to JCV or INKV-induced disease without either endosomal or cytoplasmic PRRs signaling, we crossed *Mavs*^-/-^ and *Unc93b1*.3D mice to create a *Mavs*^-/-^x*Unc93b1*.3D DKO mouse that lacked RLR and endosomal TLR signaling pathways. Inoculation of these mice with INKV and JCV resulted in 100% susceptibility to neuroinvasive disease with similar kinetics as the *Irf3*^-/-^x*Irf7*^-/-^ DKO mice for INKV and *Ifnar1*^*-/-*^ mice for JCV (Figs 7 and 6B). These results indicate that having either functional MAVS or TLR3/7/9 signaling pathway was sufficient for protection from neuroinvasive disease by JCV and INKV. These results contrast with previous results with LACV, where both pathways were required for protection [10].

### IFN responses to JCV and INKV in the periphery of immune deficient mice

The results from the weanling B6 mice time course studies identified several IFN mRNAs with increased expression in the LN at 1 dpi, including *Ifna4*, *Ifna11*, *Ifna12*, and *Ifn*β*1* mRNA in INKV, and *Ifn*β*1* mRNA in JCV. Therefore, in order to evaluate which IFNs may be responsible for the protection of JCV- and INKV-inoculated weanling mice, we compared the IFN responses to JCV and INKV between the same WT B6 mice from the previous IFN analysis that do not develop neuroinvasive disease and both DKO mouse strains (*Irf3*^-/-^x*Irf7*^-/-^ mice and *Mavs*^*-/-*^*xUnc93b*.*3D* mice; Fig 7A and 7B).

**Fig 8:**
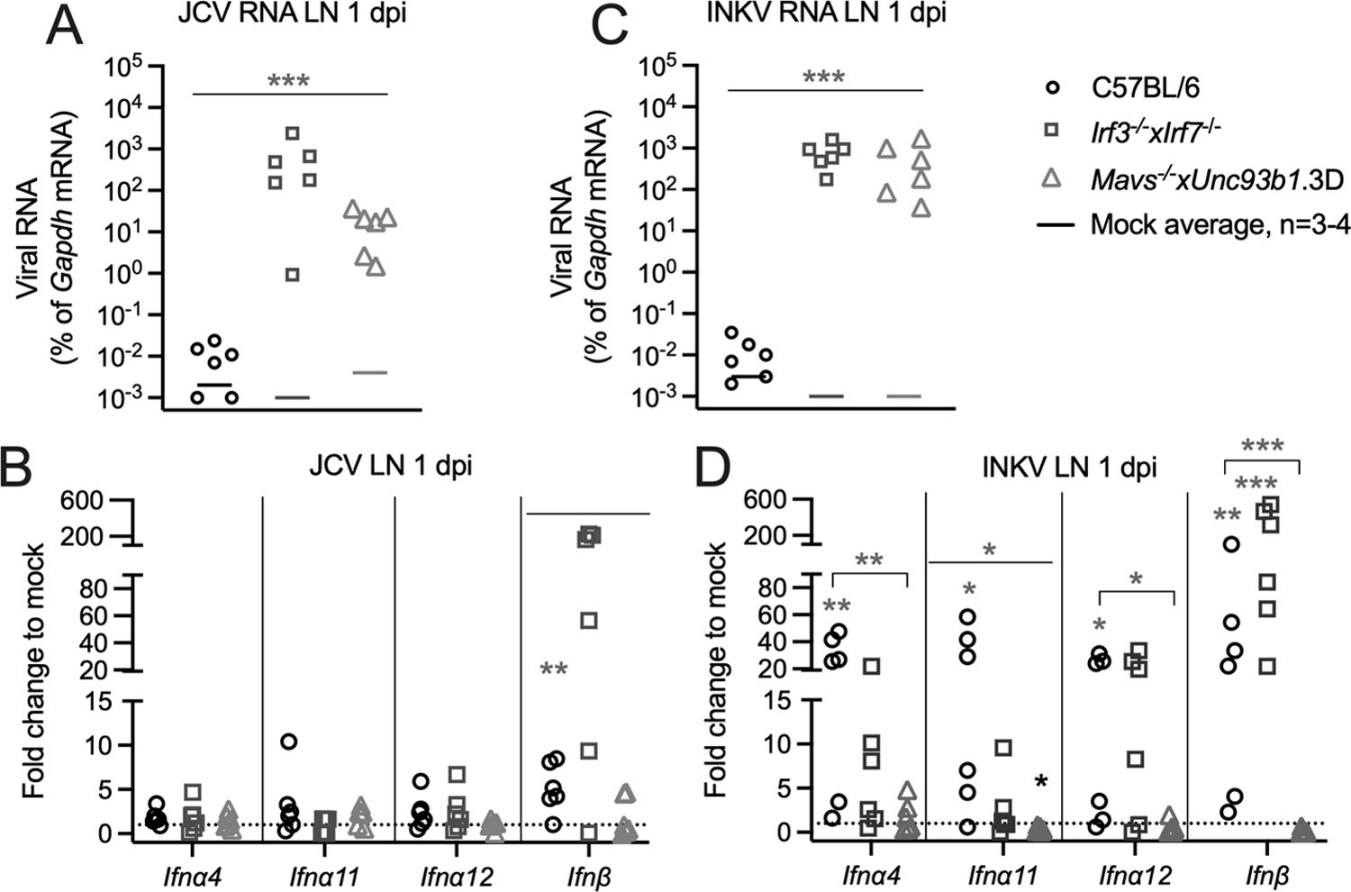
Comparison of the IFN mRNA response in LN at 1 dpi of wild type C57BL/6 mice and immune deficient DKO mice. Viral RNA was compared in the lymph nodes (LN) at 1 dpi of mice inoculated with JCV (A) or INKV (C). B,D) IFN mRNA expression was evaluated via RT-qPCR in LN at 1 dpi of JCV-inoculated mice (B) or INKV-inoculated mice (D) in B6 and DKO mice. A,C) One-way ANOVA was performed for each tissue on Log2(%gapdh) values and Dunnett’s multiple comparisons test done between B6 and DKO mice. Asterisks represent significant ANOVA and multiple comparisons p-values. B, D) There were significantly different levels in some basal *Ifn* expression between mock-inoculated B6 and DKO mouse strains, therefore Log2(fold change in %gapdh from mock) values were used to normalize expression to mock for analyses. One-sample t tests were done to analyze if the expression for each mouse strain and Ifn was significantly different from 0 (equivalent to fold change = 1 = mock). One-way ANOVA with Dunnett’s multiple comparison test between mock and the DKO strains was then run. Asterisks below the lines represent expression significantly different from mock (gray = significantly higher, black = significantly lower), and asterisks above the line represent a significant difference between B6 and one (bracketed) or both (flat line) DKO strains. Asterisks denote *p = 0.05–0.01, **p = 0.009–0.001, ***p≤0.0009, and values for the multiple comparisons are reported as the higher value from the two DKO strains. Dotted lines indicate fold change to mock = 1.

LN at 1 dpi were analyzed because they had the strongest IFN responses to JCV and INKV in the B6 mice (Fig 3A). Comparison of viral RNA levels in the LNs at 1 dpi between B6 and the two DKO strains showed that DKO mice had significantly higher viral RNA levels than the B6 mice (Fig 8A and 8C). Some differences were observed in basal IFN mRNA expression in the LN of mock-inoculated mice between WT B6 mice and the DKO strains. *Mavs*^-/-^x*Unc93b1*.3D mice had significantly higher expression of *Ifna12* mRNA and *Irf3*^*-/-*^*xIrf7*^*-/-*^ mice had significantly lower expression of *Ifn*β*1* mRNA compared to WT B6 mice (S4 Fig). Following virus infection *Irf3*^*-/-*^*xIrf7*^*-/-*^DKO mice had similar levels of IFN mRNA induction compared to B6 mice with either JCV or INKV infection, while the *Mavs*^-/-^x*Unc93b1*.3D mice had little to no IFN mRNA upregulation in response to infection (Fig 8B and 8D). The only IFN mRNA that was significantly more upregulated in B6 mice than in both DKO strains was *Ifna11* in INKV-inoculated mice (Fig 8D). The significantly higher levels of induction of *Ifna11* mRNA in the INKV-inoculated WT B6 mice compared to the DKO strains suggests that IFN*a11* could have a role in protecting mice from INKV-induced neuroinvasive disease.

### IFN and ISG responses to INKV in the brains of immune deficient mice

As INKV appears to be controlled in the brains of WT B6 mice, we next compared the IFN mRNA responses to INKV in the brains of B6, *Irf3*^*-/-*^*xIrf7*^*-/-*^ DKO and *Mavs*^*-/-*^*xUnc93b*.*3D* DKO mice to determine if a Type I IFN response in the CNS may be involved in limiting INKV infection in the brains of WT B6 mice. Comparisons were made between the same B6 mouse brains from the time course described above at 5 dpi, because this was the time point with the highest amount of INKV in the brains (Fig 1I and 1J), and DKO strains at 3 dpi, when mice succumbed to neurological disease. While our previous analysis on the IFN response in the INKV-infected mouse brains did not identify a large difference in IFN mRNA expression to mock, we compared a subset of the *Ifn*s that had increased expression in some B6 mice to determine if there were differences in IFN mRNA expression between the INKV-infected B6 and the DKO mice. Analysis of IFN and ISG mRNA expression levels in mock-inoculated mice showed higher basal levels of *Ifna4*, *Ifna12*, *Ifnε*, *Ifit1* and *Ifit2* mRNA in the brain tissue of DKO mice compared to WT B6 mice, suggesting that deficiency in innate immune signaling can heighten basal IFN and ISG mRNA expression (S4 Fig).

**Fig 9:**
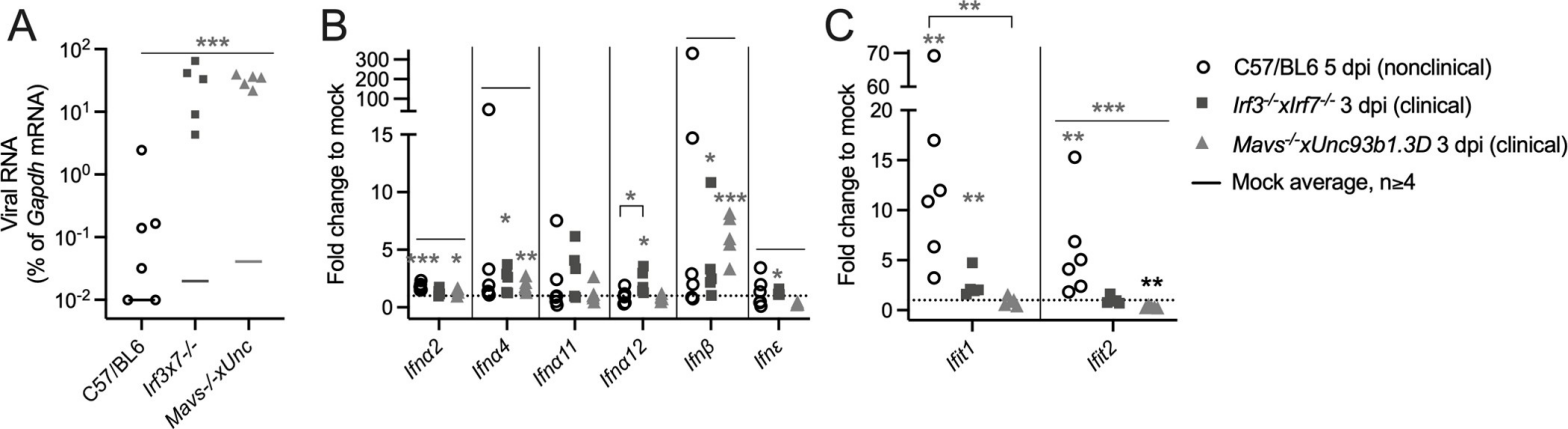
Comparison of the IFN and ISG response in brains of INKV-inoculated C57BL/6 mice versus immune deficient DKO mice. A) Viral RNA in the brains of WT C57BL/6 mice and the DKO strains, data for B6 from Fig 1I. mRNA expression levels of IFNs (B) and Ifits (C), data for B6 the same as Fig 4C and 4D. A) One-way ANOVA was performed on Log2(%gapdh) values and Tukey’s multiple comparisons test done between B6 and DKO mice. Asterisks represent significant ANOVA and multiple comparisons p-values. B & C) There were significantly different levels in basal *Ifn* and *Ifit* expression between mock-inoculated B6 and DKO mouse strains, therefore Log2(fold change in %gapdh from mock) values were used to normalize expression to mock for analyses. One-sample t tests were done to analyze if the expression for each *Ifn* and *Ifit* was significantly different from 0 (equivalent to fold change = 1 = mock average). One-way ANOVA with Dunnett’s multiple comparison test between mock and the DKO strains was then run. Asterisks below the lines represent expression significantly different from mock, and asterisks above the line represent a significant difference between B6 and one (bracketed) or both (flat line) DKO strains. Asterisks denote *p = 0.05–0.01, **p = 0.009–0.001, ***p≤0.0009, and for the multiple comparisons are reported as the higher value from the two DKO strains. Dotted lines indicate fold change to mock = 1. Nonclinical = mice with no signs of disease; Clinical = mice displaying neurological signs or other endpoint criteria.

Both DKO strains had significantly higher INKV RNA in brain tissue compared to B6 controls (Fig 9A). As in the LNs, because of the differing basal levels of IFN mRNA we again compared the levels of IFN induction from mock between B6 and the DKO strains. Interestingly, the DKO mice had IFN mRNA significantly increased from mock for *Ifna4*, *Ifna11*, *Ifn*β*1*, and *Ifnε* for the *Irf3*^*-/-*^*xIrf7*^*-/-*^ mice and *Ifna2*, *Ifna4*, and *Ifn*β*1* for the *Mavs*^*-/-*^*xUnc93b*.*3D* mice (Fig 9B). The high upregulation of IFN mRNA levels in *Irf3*^*-/-*^*xIrf7*^*-/-*^ mice correlated with the higher level of viral RNA observed in the brains of these mice (Fig 9A). However, none of the levels of upregulation were significantly different from WT B6 mice, except for *Ifna12* (Fig 9B). Overall, the induction of IFN mRNA appeared to be very similar across WT B6 and DKO mice, despite the significant difference in viral mRNA in the brains.

ISGs such as IFITs have been reported to be important for controlling virus infection in the brain [19], and we did find increased expression of *Ifit1* and *Ifit2* mRNA in the brains of INKV-inoculated B6 mice, despite the lack of significant IFN induction (Fig 4). Therefore, we compared upregulation of *Ifit1* and *Ifit2* mRNA in WT and DKO mice. Interestingly, induction of both *Ifit1* and *Ifit2* mRNA was lower in brain tissue from INKV-inoculated DKO strains compared to B6 controls (Fig 9C). This difference was significant between B6 and both DKO strains for *Ifit2* and was significantly different between B6 and *Mavs*^*-/-*^*xUnc93b*.*3D* for *Ifit1* (Fig 9C). *Ifit1* upregulation in the WT B6 mice also trended higher than in the *Irf3*^*-/-*^*xIrf7*^*-/-*^ mice, although this was not significant (Fig 9C). These trends are particularly striking given the overall similar IFN induction between B6 and DKO mice, and the much higher viral RNA levels in DKO mice. Overall these results show that the induction of ISGs in the CNS correlated with protection, not with viral RNA levels or even *Ifn* expression. The induction of ISGs in the CNS could be a critical mechanism by which the CNS controls INKV infection and prevents the development of neurological disease.

## Discussion

The results from these studies showed that differences in the ability of the CSG viruses to cause neurological disease mostly correlated with their ability to gain access to the CNS, with the exception of INKV. Previous studies have demonstrated that SSHV, TAHV, and JCV are highly neuropathogenic in mice when inoculated IN [8], indicating that once SSHV, TAHV, or JCV reach the brain they are capable of robust replication which results in neurological disease. In contrast, INKV entered the brain in most mice, but none of the INKV-inoculated mice developed neuroinvasive disease, making INKV unique among these CSG viruses in that it is neuroinvasive, but not highly neurovirulent.

Understanding why INKV does not replicate well within the CNS could provide mechanistic insight into how to prevent replication of the other more highly pathogenic CSG viruses. The amount of INKV and number of mice with detectable virus in the brain decreased from 5 to 7 dpi and INKV was undetectable by 14 dpi, suggesting that INKV is either cleared from the brain by an immune response, or INKV simply cannot establish a productive infection. Previous studies and our current results suggest it is likely a combination of both. Our previous studies have shown that even when inoculated directly IN, INKV only caused neurological disease in some mice [8]. Although all five CSG viruses were found throughout most regions of the brain, INKV-inoculated mice that developed neurological disease had far less viral antigen staining than mice infected with the other CSG viruses [8]. However, there was not a difference in cell tropism within the brains, as all CSG viruses primarily infected neurons [8]. IHC of brains from weanling B6 mice inoculated IP with LACV and INKV showed similar regions of infection in these mice as well (Table 1 and Fig 5). Additionally, INKV replicated more slowly and/or induced less cell death in human neuronal cells in vitro than the other CSG viruses [8], indicating INKV does not replicate as well in neurons as the other CSG viruses. However, we found that *Ifnar1*^-/-^, *Irf3*^*-/-*^*xIrf7*^-/-^ DKO, and *Mavs*^-/-^x*Unc93b1*.3D DKO mice inoculated with INKV all developed neurological disease (Figs 6A and 7A). Thus, a type I IFN-mediated immune response is crucial for the protection of mice from INKV neuroinvasive disease, and the lack of neuroinvasive disease is not simply due to an inability of INKV to replicate well in the brain.

We found that induction of IFN mRNA in the brains of INKV-inoculated B6 and DKO mice was similar (Fig 9B). This is in slight contrast to previous results in studies of West Nile virus in *Irf3*^*-/-*^*xIrf7*^-/-^ DKO mice, which found that in most cell types *Ifna* expression was completely abolished, while *Ifn*β expression was still induced [28]. While we did observe some increase in both *Ifna* and *Ifn*β*1* mRNA in brains of INKV-inoculated *Irf3*^*-/-*^*xIrf7*^-/-^ DKO mice, these increases were small and not observed in all mice (Fig 9B). However, we did see higher upregulation of *Ifit1* and *Ifit2* mRNA in the brains of B6 mice (Fig 9C). These findings suggest that the ISG response could be a key component in limiting INKV replication within the brain. Indeed, this is a mechanism of protection observed in vesicular stomatitis virus (VSV) infection, where localized activation of IFN in the olfactory bulb, the entry point of VSV in the brain, results in dissemination of the IFNs throughout the brain and a global induction of *Ifit2*, which prevents VSV from spreading in the brain [19,29,30]. Previous studies of LACV have shown that it enters the brain through capillary breakdown specifically in the olfactory bulb [25]. In the INKV 5 dpi brains where viral antigen was present, both brains had detectable virus in the olfactory bulb (Table 1). It is possible that INKV enters the brain in a similar manner as LACV, stimulates a localized IFN response which spreads throughout the brain resulting in global stimulation of ISGs which then prevent INKV from spreading throughout the brain and causing disease. A localized IFN response could explain why we saw such little IFN expression in brains of most INKV-infected B6 mice (Figs 4D and 9B), as the signal would be diluted in the half-brain homogenates used for RNA extraction and analysis. Because our previous studies have shown that INKV does not replicate in neurons as well as LACV [8], it is possible that the similarly modest ISG response observed in LACV and INKV (Fig 4E and 4F) is sufficient to prevent spread of the lowly neurovirulent INKV, but is overwhelmed by the highly neurovirulent LACV, resulting in the differences in disease development between them. INKV may even stimulate a better ISG response earlier than LACV, as observed by the similar ISG levels between LACV and INKV infected brains at 3 dpi, despite INKV having a lower viral load at that time point (Fig 4A and 4F). Determining why INKV replicates more slowly in neurons will be important in understanding its limited pathogenesis.

Interestingly, JCV, which has the highest sequence identity to INKV of these CSG viruses [31], does not have the same replication issues as INKV within the CNS, as JCV is highly neurovirulent in mice when inoculated IN [8]. The finding that JCV does not enter the brains of WT weanling mice indicates that a lack of neuroinvasion is the limiting factor for JCV-induced neurological disease. While we have previously shown that LACV induces BBB breakdown which facilitates LACV entry into the brain [25], the viral factors that mediate this damage are completely unknown. It is possible that JCV may lack the functional viral factors involved in neuroinvasion that LACV, and presumably the other neuroinvasive CSG viruses, has. However, JCV did induce neurological disease in mice with severely inhibited IFN signaling. Therefore, it may be more likely that JCV encodes viral factors that are less efficient at inducing BBB breakdown compared to the other CSG viruses, and JCV is controlled by the IFN response in the periphery prior to inducing BBB breakdown. The lack of sufficient IFN signaling in the *Ifnar1*^*-/-*^, *Irf3*^*-/*^*-xIrf7*^*-/-*^ and *Mavs*^*-/-*^*xUnc93b1*.3D mice, which all developed neurological disease, may allow JCV to replicate so abundantly that it can overcome this deficiency and induce BBB breakdown. Alternatively, JCV may enter brains by an entirely different mechanism compared to LACV. An alternative mechanism of neuroinvasion may help to explain why JCV has the most divergent neuropathogenesis between humans and the mouse model. Unlike the other CSG viruses which cause disease primarily in children and weanling mice, JCV primarily induces neuroinvasive disease in adult humans [1]. However, JCV does not induce neuroinvasive disease in adult mice. The interactions between host and JCV required for JCV neuroinvasion may not be fully recapitulated between adult mice and adult humans. Further studies to elucidate the differences in molecular mechanisms of neuroinvasion of the CSG viruses are needed to address this.

There are also likely underlying genetic differences in viral factors that mediate differences between LACV and INKV neuropathogenesis. The only known virulence factor in LACV is the Nonstructural protein S (NSs), which has been shown to be an IFN antagonist by degrading host RNA polymerase II [32–34]. However, NSs cannot be the only virulence factor for LACV, as most WT mice infected with a LACV NSs deletion virus still developed disease [33]. The function of NSs is uncharacterized in INKV and the other CSG viruses, however, they are all predicted to encode the NSs protein. It is unknown if NSs contributes to the differences observed in pathogenesis between CSG viruses, and additional studies to investigate this are needed.

Our results also demonstrate that fewer components of the innate immune response are required to control INKV and JCV infection compared to LACV. Previous studies showed that for LACV, wild-type adult B6 mice did not develop neuroinvasive disease, but >75% of both adult *Mavs*^*-/-*^ mice and *Unc93b1*.3D mice (deficient in TLR3/7/9 signaling) were susceptible to LACV neuroinvasive disease. Therefore, functional signaling through both MAVS and TLR3/7/9 are required to protect adult mice from LACV neuroinvasive disease [10,35]. In contrast, in the current study, only about 25% of *Mavs*^*-/-*^ mice and 10% of *Unc93b1*.3D mice were susceptible to neuroinvasive disease following INKV or JCV inoculation (Fig 6), suggesting that having either functional MAVS signaling or TLR3/7/9 signaling was sufficient to protect the majority of mice from JCV and INKV neuroinvasive disease. However, *Mavs*^*-/-*^x*Unc93b1*.3D DKO mice deficient in both pathways were highly susceptible to both INKV- and JCV-induced neuroinvasive disease (Fig 6). These results clearly indicate that the ability of the host to protect against INKV and JCV neuroinvasive disease requires a less robust immune response than that needed to protect against LACV neuroinvasive disease, and the immune pathway components involved in protection were shared for JCV and INKV. The ability of either arm of the innate immune response to control INKV and JCV infection may partially explain why weanling mice, which have a weaker IFN response to virus infection than adults [10], are still able to control INKV and JCV, but not LACV.

Despite JCV and INKV requiring similar innate immune components for protection, the IFN mRNA response to these two viruses were quite disparate, with JCV having a very modest response and INKV producing high levels of IFN mRNA in the periphery that was often higher than the other viruses (Fig 3). This increased IFN response to INKV was not due to higher viral RNA loads in the periphery. In fact, neither JCV or INKV had viral levels significantly different from mock in LN or spleens at any time point, whereas each of the neuropathogenic viruses (LACV, SSHV, and TAHV), had at least one day in the LN with significantly elevated levels of viral RNA (Fig 2). Despite the robust IFN response, INKV was still capable of entering the brains of mice, indicating that the peripheral type I IFN response couldn’t completely control INKV infection. However, very little infectious virus was observed in the periphery of INKV-inoculated WT B6 mice, but high levels of infectious INKV was observed in *Ifnar1*^*-/-*^ mice (Fig 6C and 6D), suggesting the robust IFN response to INKV observed in the periphery of B6 mice may limit the amount of INKV that is able to enter the brain. Why JCV and INKV have the same requirements for protection and similarly low amounts of viral RNA in the periphery, but differences in IFN induction and neuroinvasiveness is unclear, and further studies to elucidate this are needed.

The current studies were unable to definitively identify a type I IFN or a group of type I IFNs whose expression was associated with reduced risk for neurological disease. Across all CSG viruses, the IFN mRNA that was most consistently upregulated was *Ifn*β*1* (Figs 3 and S1). However, when IFN responses to JCV and INKV in the LN at 1 dpi were compared between disease-resistant WT B6 mice and disease-susceptible DKO strains, *Ifn*β*1* was highly expressed in most *Irf3*^*-/-*^*xIrf7*^*-/-*^ mice as well (Fig 7B and 7D), so it likely does not play a significant role in mediating protection from these viruses. Interestingly, *Ifna11* was higher at 1 dpi in LNs of B6 mice compared to the DKO mice during both JCV and INKV infection, although the difference was only significant for INKV (Fig 7B and 7D). Previous studies have shown *Ifna11* has antiviral activity against a variety of viruses, and controls retrovirus infections by activating natural killer cells [36,37]. It is possible that for both JCV and INKV, the modest *Ifna11* response may help control virus replication in the periphery, although this does not prevent INKV from gaining access to the CNS. Targeting deficiencies in different IFN alpha genes or treatment with specific IFNs may help determine which IFNs are critical for protection against JCV and INKV.

Overall, the results from these studies show that the CSG viruses have different neuroinvasive abilities that can be independent from their ability to cause neurological disease. Innate immune responses, particularly the IFN response, appear to play a critical role in the protection of mice from CSG virus neuroinvasive disease. However, the signaling pathways and levels of IFN required for protection appears to vary between CSG viruses. Understanding these differences will provide insights for drug and vaccine development for the CSG viruses.

## Methods

### Ethics statement

All mouse experiments were performed in full accordance with the National Institutes of Health Guidelines and approved by the Rocky Mountain Laboratories (RML) Animal Care and Use Committee (Hamilton, MT) under protocols 2016-061-E and 2019-051-E.

### Cells and viruses

Virus stocks of LACV (human 1978), SSHV (1976), TAHV (92 Bardos), JCV (61V2235), and INKV (SW AR 83–161) used in this study were previously described in [8]. Vero cells were grown in Dulbecco modified Eagle medium (DMEM, Gibco) supplemented with 10% fetal bovine serum (Atlas Biologicals) and 1% penicillin/streptomycin solution (Gibco). Vero cells were passaged and plated via chemical dissociation with 0.25% trypsin.

### Mouse strains and inoculations

Wild-type C57BL/6 (B6) mice were used for time point experiments, and all immune deficient mice were maintained on the B6 background. Immune deficient strains used were knockout (KO) mice which lacked the Type I IFN receptor (*Ifnar1*^*-/-*^ mice, acquired from The Jackson Laboratory), mice with double KO (DKO) of interferon regulatory factor (IRF) 3 and IRF7 (*Irf3*^*-/-*^*xIrf7*^*-/-*^ DKO mice [28], provided by Michael Diamond); mice with the innate signaling adaptor protein MYD88 KO (*Myd88*^*-/-*^ mice [38], acquired from the Mutant Mouse Regional Resource Center); mice with a missense allele of *Unc93b1* resulting in abolished signaling through endosomal Toll-like receptors (TLR) 3, 7, and 9 (*Unc93b1*.3D mice [39], acquired from the Mutant Mouse Regional Resource Center); mice with a mitochondrial antiviral signaling (MAVS) protein KO (*Mavs*^*-/-*^ mice, acquired from The Jackson Laboratory). *Mavs*^*-/-*^*xUnc93b1* 3D DKO mice were made at RML by crossing *Mavs*^*-/-*^ mice with *Unc93b1*.3D mice, genotyping progeny in-house, and crossing heterozygous/homozygous progeny generations until a double homozygous knockout colony was established.

All inoculations were done intraperitoneally (IP) in weanling mice between 21–23 days of age. Phosphate-buffered saline (PBS) was used to dilute viruses to 10^5^ plaque forming units (PFU) in a volume of 200 μl for inoculation. Isoflurane was used to anesthetize mice for inoculation. Mice were checked twice daily following inoculation for clinical signs. Endpoint criteria included neurological signs such as ataxia, limb weakness, tremors, circling, and seizures, and mice that were moribund or had limited movement that inhibited access to food or water. Any mouse showing neurological signs or other endpoint criteria was immediately humanely euthanized. For time course experiments, a subset of mice were humanely euthanized and tissues removed at 1, 3, 5, 7, and 14 days post inoculation (dpi). For both time point experiments and survival experiments, any mice that displayed neurological signs or other endpoint criteria, regardless of day, were immediately humanely euthanized. For time course experiments, blood was collected via cardiac puncture in heparin from mice under deep anesthesia with isoflurane, then mice were perfused transcardially with heparin-saline. Spleens, inguinal lymph nodes, brains, kidneys, livers, lungs, hearts, and blood in heparin were taken from some or all mice. Tissues used for RNA analysis and plaque assays, except blood, were immediately flash-frozen in liquid nitrogen, then subsequently stored at -80°C until use. For brains used in histology analysis, brains were cut saggitally and one saggital half was fixed in 10% formalin. Plasma was separated from the whole blood via centrifugation at 5,000xg for 10 minutes, then stored at -80°C. Mice from survival curve experiments that did not develop clinical disease were humanely euthanized by 30 dpi. For the time course experiments in B6 mice, n = 6 for each virus and time point of 1, 3, 5, and 7 dpi, except n = 4 for LACV at 6 dpi, no LACV-inoculated mice survived to 7 dpi, n = 5 for SSHV at 5 dpi, two mice from the SSHV-inoculated cohort designated for 7 dpi developed neurological signs at 6 dpi and had to be euthanized, so n = 2 for 6 dpi, n = 4 for 7, and for TAHV, one mouse in the designated 5 dpi cohort developed neurological disease at 4 dpi and had to be euthanized, so n = 1 for 4 dpi and n = 5 for 5 dpi, n = 7 for mock, and only INKV was evaluated at the 14 dpi timepoint, with n = 6. For the survival curves of immune deficient strains inoculated with JCV and INKV, n = 6 for INKV and n = 5 for JCV in C57BL/6 mice, n = 9 for INKV and n = 7 for JCV in *Ifnar1*^-/-^ mice, n = 8 for INKV and JCV in *Irf3*^*-/-*^*xIrf7*^-/-^ mice, n = 5 for INKV and n = 8 for JCV in *Myd88*^-/-^ mice, n = 11 for INKV and n = 10 for JCV in *Unc93b1*.3D mice, n = 14 for INKV and n = 10 for JCV in *Mavs*^-/-^ mice, and n = 9 for INKV and n = 10 for JCV in *Mavs*^-/-^x*Unc93b1*.3D mice. For time point analysis of the DKO strains, n = 4 for mock *Irf3*^*-/-*^*xIrf7*^-/-^ and *Mavs*^-/-^x*Unc93b1*.3D mice. n = 6 for INKV at 1 dpi, and JCV at 1 and 3 dpi for both DKO strains, and n = 5 for INKV at 3 dpi in both DKO strains.

### Plaque assays for infectious virus

For analysis of infectious virus from tissues via plaque assay in Vero cells, ½ brain, spleen, both kidneys, heart, liver, and lung were weighed and homogenized in 500 ul of serum-free DMEM or 1x PBS in 2 ml cryovials (Sarstedt) containing 2.3 mm Zirconia/Silica beads (Fisher) on a bead beater at 5300 rpm for 25 seconds. Samples were then clarified at 5000 x g for 10 minutes, and the clarified supernatant transferred to a new tube. Vero cells were plated the previous day in 24-well plates (Corning) at a density of ~1.3 x 10^5^ cells per well. The clarified tissue homogenate supernatants and plasma samples were serially diluted 10-fold in DMEM supplemented with 2% FBS and penicillin-streptomycin. Media was removed from plates, and 200ul of serially diluted sample was plated per well and plates were incubated at 37°C for one hour. After incubation, 0.5 ml of MEM (Gibco) with 1.5% carboxymethyl cellulose (CMC) was overlayed per well, and plates were returned to the 37°C incubator. After 3 days for INKV and SSHV, and 5 days for LACV, TAHV, and JCV, plates were fixed with 10% formaldehyde (Sigma), stained with 0.35% crystal violet, rinsed, air-dried, and plaques counted for each sample and dilution. Sample titers were calculated as PFU/mg tissue or PFU/ml plasma.

### RNA isolations and RT-qPCR

RNA was extracted from ½ brains, spleens, and inguinal lymph nodes following a Trizol and chloroform protocol, and all centrifugation was done at 4°C. Brains were homogenized in 1.5 ml Trizol and spleens and lymph nodes were homogenized in 1 ml Trizol in bead tubes (Sarstedt) containing 2.3 mm Zirconia/Silica beads (Fisher) on a bead mill for 5200 rpm for 20 seconds x 2 with 5 seconds of dwell. Homogenized samples were transferred to 1.5 ml microcentrifuge tubes, 200 ul chloroform added to each sample, shaken vigorously, and centrifuged at 12,000 x g for 15 minutes. The aqueous phase was transferred to a new tube and RNA precipitated with 600 ul isopropanol for ~1 hour, then centrifuged at 12,000 x g for 10 minutes to pellet the RNA. The supernatant was removed from the tube and the pellet was washed in 1 ml 70% EtOH, vortexed, and centrifuged at 7,600 x g for 5 minutes. The supernatant was removed, pellets air-dried, resuspended in 50 ul (spleens and lymph nodes) or 100 ul (brains) nuclease-free water, incubated at 55°C for 10 minutes, then stored at -80°C. Samples were then DNase treated using 5 ul brain sample, 20 ul spleen sample, and 50 ul lymph node sample following DNase I kit instructions (Ambion DNase). DNase-treated samples were then cleaned up with the Zymo RNA cleanup kit following manufacturer’s instructions, with the exception that RNA wash steps were performed with 600 ul Wash Buffer and RNA was eluted in 50 ul warm NF H_2_O. cDNA was synthesized from the cleaned up RNA using the BioRad iScript cDNA synthesis kit, following kit instructions. The following primer sets were used in RT-qPCR. For viral specific RNA: LACV in all tissues: forward 5’-ATTCTACCCGCTGACCATTG-3’, reverse 5’-GTGAGAGTGCCATAGCGTTG-3’; SSHV in brains: forward 5’-AGCATGATCAAAACGGAGGC-3’, reverse 5’-CATGCCAATCAGACACCAGC-3’; SSHV in lymph nodes and spleens: forward 5’-ACTCACAGCAGACAAGTGGA-3’, reverse 5’-TGATCTGGCAGTGTCGCTTA-3’; TAHV in all tissues: forward 5’-AGGTCCTACATTGCCGTTCA-3’, reverse 5’-TGGTCTACAGGTGCTAGCTC-3’; JCV in all tissues: forward 5’-TATGGTTCCCCGGTAGTGTG-3’, reverse 5’-TAACATGGTGCTTCTCGTGC-3’; INKV in brains: forward 5’-AGTCCAAGATAAAGCCCCAGA-3’, reverse 5’-TCATGTTAGCCTGGCATCCA-3’; and INKV in lymph nodes and spleens: forward 5’-AGTCCAAGATAAAGCCCCAGA-3’, reverse 5’-TCATGTTAGCCTGGCATCCA-3’. The following IFN and ISG primer sets were used: *Ifna1*: forward 5’-AGCCTTGACACTCCTGGTAC-3’, reverse 5’-AGCCTTCTTGATCTGCTGGG-3’; *Ifna2*: forward 5’-CCCTTTCTCTCCTGCCTGAA-3’, reverse 5’-TAAGATCTCGCAGCACAGGG-3’; *Ifna4*: forward 5’-CTGCTGGCTGTGAGGACATA-3’, reverse 5’-AGGAAGAGAGGGCTCTCCAG-3’; *Ifna6*: forward 5’-GGTTTTGGTGGTGTTGAGCT-3’, reverse 5’-GTACCAGGAGTGTCAAGGCT-3’; *Ifna9*: forward 5’-TCATCTGCTGCTTGGAATGC-3’, reverse 5’-AGTTCCTTCATCCCGACCAG-3’; *Ifna11*: forward 5’-ACCTGTTCTCTAGGATGCGA-3’, reverse 5’-TTAGGCAGGAAAGAGGGGTG-3’; *Ifna12*: forward 5’-CCTAGAGGGGAAGGTTCAGG-3’, reverse 5’-AGCTCATCACTAGCAGGGTC-3’; *Ifn*β*1*: forward 5’-AGCACTGGGTGGAATGAGAC-3’, reverse 5’-TCCCACGTCAATCTTTCCTC-3’; *Ifnε*: forward 5’-CCTTCAGCAGATCTTCACGC-3’, reverse 5’-TGACTCCACGTATTCCAGCT-3’; *Ifnκ*: forward 5’-AAACGCCGTCTCCTATCGTA-3’, reverse 5’-CCCGATCTGATACGTTCCCA-3’; *Ifi202b*: forward 5’-GGCAATGTCCAACCGTAACT-3’, reverse 5’-CAGGAGAGGCTTGAGGTTGA-3’; *Ifit1*: forward 5’-TGCTGTTTTGGACTCCTGTG-3’, reverse 5’-GGACATTAGCAAAGGGTGGA-3’; *Ifit2*: forward 5’-CCTGGATCAAGAATGGGCTA-3’, reverse 5’-CATCCCACGATCCAGAAACT-3’; and *mMx1*: forward 5’-TCTGGAAGCACTGTCTGGAG-3’, reverse 5’-ACTTTGCCTCTCCACTCCTC-3’, and the housekeeping gene Gapdh: forward 5’-AACGACCCCTTCATTGAC-3’, reverse 5’-TCCACGACATACTCAGCA-3’. mRNA analysis was performed as previously described [40]. Briefly, qPCR reactions were set up in triplicate in 384-well plates and run on an ABI 7900 machine (Applied Biosystems). The percentage difference in Ct values with the housekeeping gene Gapdh were calculated (ΔCt = (Ct *Gapdh)*–(Ct gene of interest)) for each sample (“% gapdh”). Fold changes of host response genes were calculated comparing the % gapdh values of mock controls with infected samples. Samples from cDNA synthesis reactions run without reverse transcriptase and water-only samples were run as controls.

### Immunohistochemistry

A saggital half of each brain for IHC studies was fixed in 10% neutral buffered formalin, washed in 1x PBS, and cryoprotected in 20% sucrose, then 30% sucrose in PBS. Tissues were embedded in Tissue-Tek O.C.T Compound (Sakura) and frozen on dry ice or in liquid nitrogen. Using a Leica CM3050S cryostat, sections were cut at 10-μm and mounted on slides. Sections were washed in 1x PBS, incubated 30 minutes in Blocking Buffer (1x PBS with 5% normal donkey serum, 0.1% triton-X, and 0.3M glycine), and primary antibodies for virus (in-house generated hyperimmune serum raised in C57BL/6 mice against LACV, diluted 1:200), anti-CD3 (Invitrogen, 14-0032-85, diluted 1:200), and anti-IBA1 (Wako, 019–19741, diluted 1:250) were diluted in Blocking Buffer and applied to tissues at 4°C overnight. Tissues were washed in 1x PBS, then incubated with secondary antibodies (Life Technologies, donkey anti-mouse Alexa Fluor 647, donkey anti-rat Alexa Fluor 488, and donkey anti-rabbit Alexa Fluor 594 all diluted at 1:500) in 1x PBS for one hour at room temperature. Sections were then washed in 1x PBS, nuclei stained with Hoechst 33342 (Invitrogen, H3570, diluted 1:1000), and mounted on slides with ProLong Gold anti-fade reagent (Invitrogen P36930) and cover-slipped. A single full brain section from each mouse was scanned using a Zeiss Axio Scan.Z1 with the 20x objective.

IBA1+ microglial cell activation was defined by the prescene of IBA1+ cells in the brain parenchyma that had retracted and thicken processes with intense staining. These microglia cells were distinct in morphology and location from infiltrating monocyte/macrophages found in perivascular regions.

### Statistical methods

All analyses were performed in PRISM version 9 (GraphPad). For all analyses of qRT-PCR gene expression data, the %gapdh values were Log2 transformed. For gene expression analysis comparing WT C57BL/6 mice with the DKO strains mock *Irf3*^*-/-*^*xIrf7*^-/-^ and *Mavs*-^/-^x*Unc93b1*.3D, basal levels of *Ifns* and ISGs in mock-inoculated mice were compared via one-way ANOVA and were determined to be significantly different between mouse strains. Therefore, for these analyses, Log2(fold change in %gapdh from mock) values were used to normalize expression to mock for each strain in order to compare expression across strains. For these, a one-sample t-test was performed for each strain and gene to determine if expression was significantly different from 0, because 0 = (fold change = 1) = mock average. Analysis by t-test or one-way ANOVA are specified for each figure, and multiple comparisons tests were performed via Dunnett’s or Sidak’s multiple comparisons test, as specified in the figure legends. Results were reported as significant if p<0.05.

## Supporting information

S1 Fig IFN response to CSG viruses in the periphery. IFN mRNA expression of a subset of IFNs in the A) lymph nodes at 3 dpi, B) lymph nodes at 4/5 dpi for TAHV, and C) spleens at 1 dpi. Individual symbols correspond to the same IFN across graphs. Dotted lines indicate fold change = 1 to mock average. For all graphs, fold change to mock is plotted on the left axis, viral RNA plotted on the right axis. One-way ANOVA analyses were performed on Log2(%gapdh) with Dunnett’s multiple comparison test performed between mock and each sample.
(TIF)

S2 Fig IFN and ISG mRNA response in brain tissue of INKV-infected mice at 7 dpi. A) IFN mRNA expression and B) ISG mRNA expression. Individual symbols represent individual mice. Dotted lines indicate fold change = 1 to mock average. One-way ANOVA on Log2(%gapdh) values with Dunnett’s multiple comparison test performed between mock and each IFN and ISG mRNA revealed none of the IFN or ISG expression levels were significantly different from mock.
(TIF)

S3 Fig T cell infiltration in the olfactory bulb of LACV, INKV or mock-infected mice. Regions from the olfactory bulb of (top to bottom), 3 dpi LACV, 5 dpi LACV, 3 dpi INKV, 5 dpi INKV and mock-inoculated animals were immunohistochemically labeled for the T cell-specific marker CD3, virus (white) and cell nuclei (blue). All three labels are shown in the images in the left column. The images in the right column show only CD3 and cell nuclei to more clearly demonstrate labeled T cells. Notice only virus-infected sections at 5 dpi (second and forth image in each column) contain appreciable numbers of labeled T cells. The white scale bar in the top right image represents 50μm and corresponds to all other images.
(TIF)

S4 Fig Basal IFN and ISG levels in tissues from WT B6 and double knockout mice. Basal levels of IFN and ISG expression in mock-inoculated mice in A) lymph nodes, and B) brains for WT C57BL/6 and *Irf3*^*-/-*^ x *Irf7*^*-/-*^ and *Mavs*^*-/-*^ x *Unc93b1*.3D DKO mice. One-way ANOVA on Log2(%gapdh) values with Dunnett’s multiple comparison test performed between C57BL/6 and each DKO strain. Asterisks above the line represent a significant difference between C57BL/6 and one (bracketed) or both (flat line) DKO strains. Asterisks denote *p = 0.05–0.01, **p = 0.009–0.001, ***p≤0.0009, and for the multiple comparisons are reported as the higher value from the two DKO strains.
(TIF)
