# Supplementary figures and images for: Differences in neuroinvasion and protective innate immune pathways between encephalitic California Serogroup orthobunyaviruses
Source: PLoS Pathog. 2022 Mar 4;18(3):e1010384. doi: 10.1371/journal.ppat.1010384 (PMC8926202; doi:10.1371/journal.ppat.1010384)

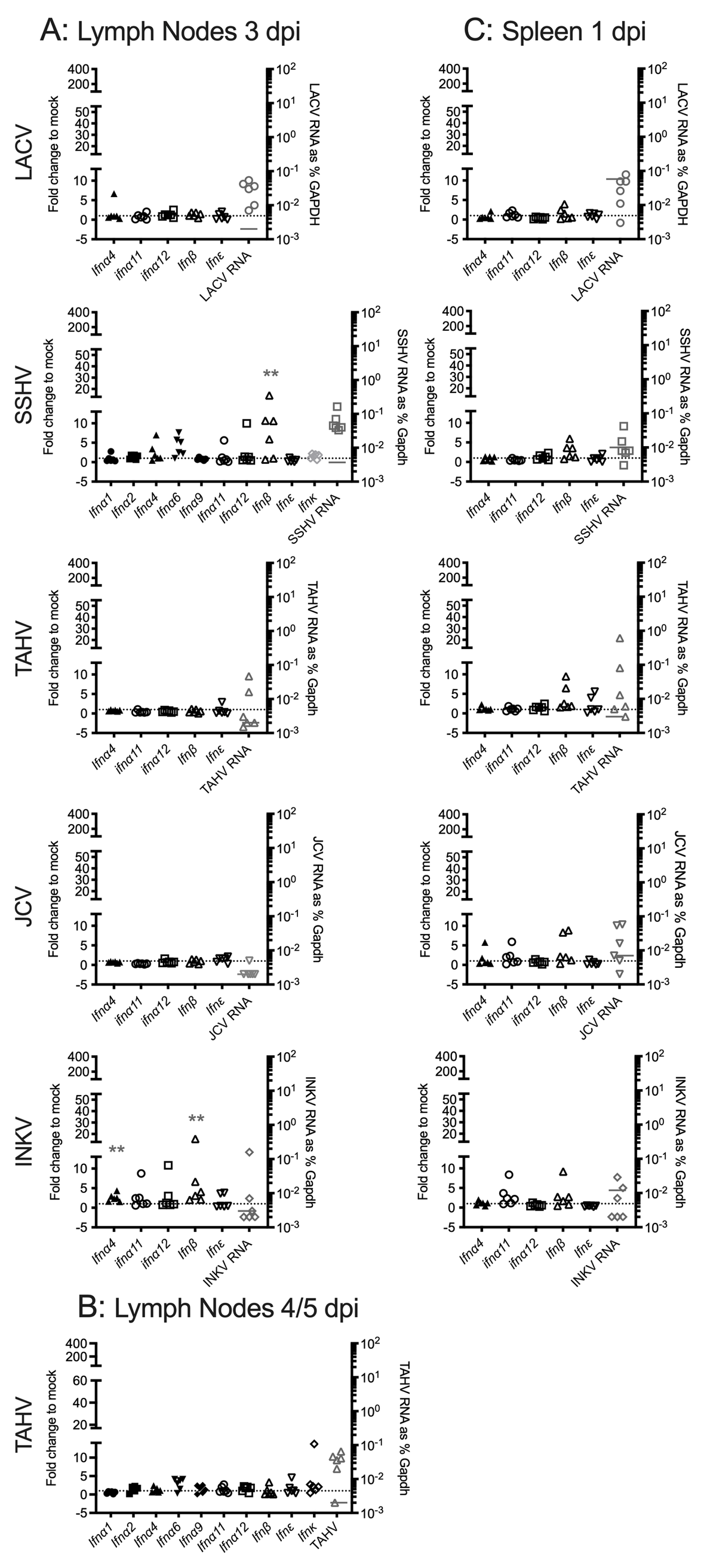

Supplement: S1 Fig — IFN mRNA expression of a subset of IFNs in the A) lymph nodes at 3 dpi, B) lymph nodes at 4/5 dpi for TAHV, and C) spleens at 1 dpi. Individual symbols correspond to the same IFN across graphs. Dotted lines indicate fold change = 1 to mock average. For all graphs, fold change to mock is plotted on the left axis, viral RNA plotted on the right axis. One-way ANOVA analyses were performed on Log2(%gapdh) with Dunnett’s multiple comparison test performed between mock and each sample. (TIF) [file ppat.1010384.s001.tif]

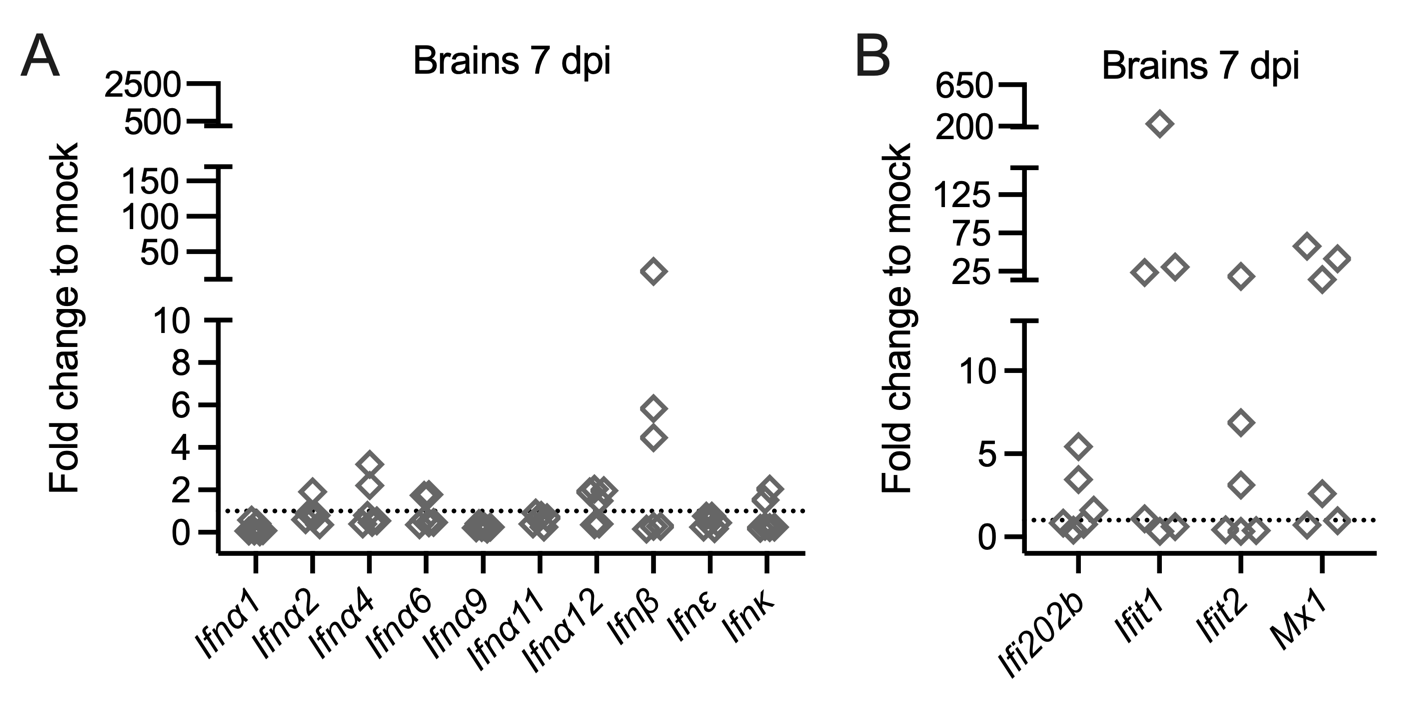

Supplement: S2 Fig — A) IFN mRNA expression and B) ISG mRNA expression. Individual symbols represent individual mice. Dotted lines indicate fold change = 1 to mock average. One-way ANOVA on Log2(%gapdh) values with Dunnett’s multiple comparison test performed between mock and each IFN and ISG mRNA revealed none of the IFN or ISG expression levels were significantly different from mock. (TIF) [file ppat.1010384.s002.tif]

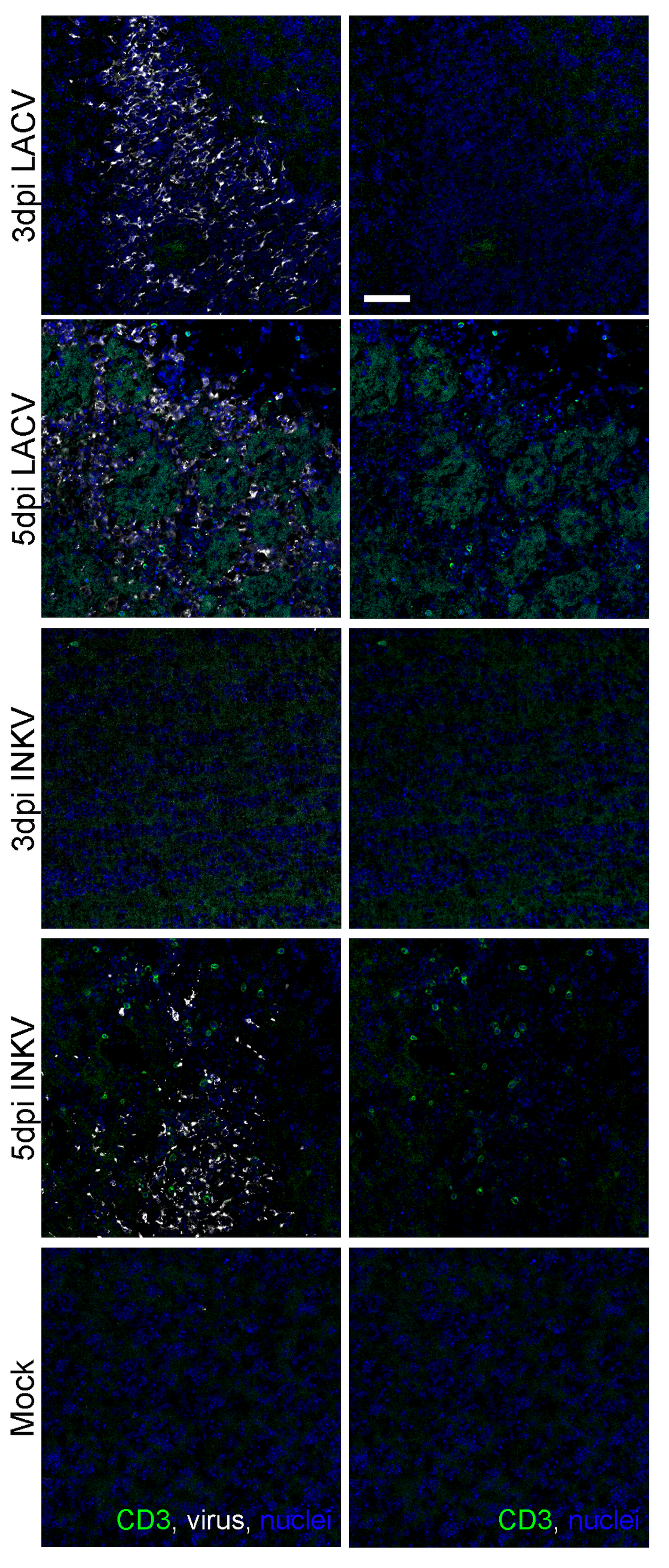

Supplement: S3 Fig — Regions from the olfactory bulb of (top to bottom), 3 dpi LACV, 5 dpi LACV, 3 dpi INKV, 5 dpi INKV and mock-inoculated animals were immunohistochemically labeled for the T cell-specific marker CD3, virus (white) and cell nuclei (blue). All three labels are shown in the images in the left column. The images in the right column show only CD3 and cell nuclei to more clearly demonstrate labeled T cells. Notice only virus-infected sections at 5 dpi (second and forth image in each column) contain appreciable numbers of labeled T cells. The white scale bar in the top right image represents 50μm and corresponds to all other images. (TIF) [file ppat.1010384.s003.tif]

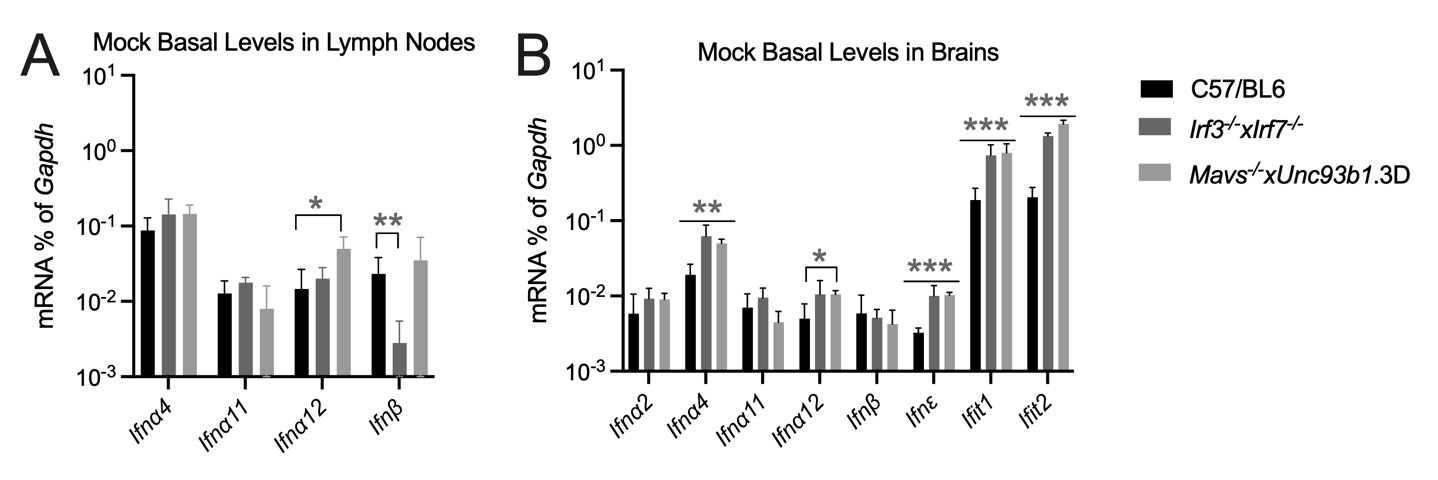

Supplement: S4 Fig — Basal levels of IFN and ISG expression in mock-inoculated mice in A) lymph nodes, and B) brains for WT C57BL/6 and Irf3-/- x Irf7-/- and Mavs-/- x Unc93b1.3D DKO mice. One-way ANOVA on Log2(%gapdh) values with Dunnett’s multiple comparison test performed between C57BL/6 and each DKO strain. Asterisks above the line represent a significant difference between C57BL/6 and one (bracketed) or both (flat line) DKO strains. Asterisks denote *p = 0.05–0.01, **p = 0.009–0.001, ***p≤0.0009, and for the multiple comparisons are reported as the higher value from the two DKO strains. (TIF) [file ppat.1010384.s004.tif]
